# Mathematical Models for Unstable Quantum Systems and Gamow States

**DOI:** 10.3390/e24060804

**Published:** 2022-06-08

**Authors:** Manuel Gadella, Sebastián Fortín, Juan Pablo Jorge, Marcelo Losada

**Affiliations:** 1 Departamento de Física Teórica, Atómica y Optica, Universidad de Valladolid, Paseo Belén 7, 47011 Valladolid, Spain; 2 CONICET, Universidad de Buenos Aires, Buenos Aires C1053 CABA, Argentina; sfortin@conicet.gov.ar; 3 Facultad de Filosofía y Letras, Universidad de Buenos Aires, Puan 480, Buenos Aires C1053 CABA, Argentina; jorgejpablo@gmail.com; 4 Instituto de Filosofía, Universidad Austral, Mariano Acosta 1611, Pilar B1629 WWA, Argentina; 5 Facultad de Matemática, Astronomía, Física y Computación, Universidad Nacional de Córdoba, Av. Medina Allende s/n, Córdoba 5000, Argentina; marcelolosada@yahoo.com

**Keywords:** unstable quantum systems, Gamow vectors, rigged Hilbert space, Gamow functionals, coherent Gamow states, intrinsic irreversibility and Loschmidt echo

## Abstract

We review some results in the theory of non-relativistic quantum unstable systems. We account for the most important definitions of quantum resonances that we identify with unstable quantum systems. Then, we recall the properties and construction of Gamow states as vectors in some extensions of Hilbert spaces, called Rigged Hilbert Spaces. Gamow states account for the purely exponential decaying part of a resonance; the experimental exponential decay for long periods of time physically characterizes a resonance. We briefly discuss one of the most usual models for resonances: the Friedrichs model. Using an algebraic formalism for states and observables, we show that Gamow states cannot be pure states or mixtures from a standard view point. We discuss some additional properties of Gamow states, such as the possibility of obtaining mean values of certain observables on Gamow states. A modification of the time evolution law for the linear space spanned by Gamow shows that some non-commuting observables on this space become commuting for large values of time. We apply Gamow states for a possible explanation of the Loschmidt echo.

## 1. Introduction, Motivation and General Considerations

Most elementary particles, as well as atoms in excited states and some molecules, are quantum unstable systems. This means that they split into subsystems, as in the case of elementary particles, or they go to a state of minor energy with the emission of one or more photons, as in the case of excited atoms. The peculiarity of quantum unstable systems is that they are quantum systems that decay.

The concept of quantum resonance comes after the notion of resonance scattering. Let us briefly describe the latter: In resonance scattering, a quantum system, like a particle, evolves freely until it enters an interaction region, in which the systems remains a time substantially bigger than the time it would remain if the interaction should not exist. After this lapse of time, the system leaves the interaction region and evolves freely again. The situation may be describe by the use of two Hamiltonians, $H_{0}$, providing the free evolution of the system, and the *total* Hamiltonian, $H=H_{0}+V$, where *V* denotes the potential responsible for the interaction. Thus, in order to have a resonance, we require a Hamiltonian pair, $\left\{H_{0},H=H_{0}+V\right\}$.

In the following paper, we are considering the situation as arises in standard non-relativistic quantum mechanics. Within this context, a quantum unstable system would be described by a resonant quantum state inside the interaction region and by its decay as it escapes to the region where the state evolves freely again, ignoring the process of the resonance creation [1]. Thus, we identify resonances with quantum unstable states, at least within non-relativistic quantum mechanics.

There are several definitions of resonances, from the physical or mathematical points of view, which are not always equivalent in all cases. Let us list some of the most relevant definitions that constitute the standard bibliography on the subject.

1. Physical definitions (see [1,2,3,4,5]).
  - Resonances appear in scattering processes as high bumps on the cross section. These bumps are characterized by two parameters: the resonance energy $E_{R}$ and the width of the bump, Γ, for which its inverse is proportional to the mean life. Generally speaking, $E_{R}$ is the difference in energy between the decaying system and the products of the decay [5].
  - The presence of resonances is also detected by *large time delays*. This is the difference in the time that one particle stays in the interaction region with or without the interaction [6].
  - Sudden change of the phase shift $\delta_{\ell }\left(E\right)$ around the resonance energy $E_{R}$.
  - Let us consider the wave function of the decaying state ψ(E) in the energy representation. If its amplitude |ψ(E)| has a Lorenztian shape,
    (1) $${\left|\psi \left(E\right)\right|}^{2}\approx N\frac{\Gamma }{{\left(E-E_{R}\right)}^{2}+\Gamma^{2}/4},$$
    then the wave function ψ(E) is the quantum state for a resonance with resonance energy $E_{R}$ and width Γ [7,8,9]. The shape of the state ψ(E) is the necessary and sufficient condition for this state to decay exponentially at all times. However, the semiboundedness property of quantum non-relativistic Hamiltonians prohibits the distribution (2). As a consequence, this exponential decay may be only an approximation at best. We already know that, for very short and very long times, serious deviations of the exponential decay emerge [5]. The exponential decay may be a good approximation for intermediate times, although deviations are not easy observable [10,11].
2. Mathematical definitions.
  - The scattering matrix *S* (also called scattering operator or *S*-operator) is a very useful tool in quantum scattering theory. This is an operator that acts on the freely evolving input state and gives as a result the freely evolving output state, $\psi^{\mathrm{out}}=S\psi^{\mathrm{in}}$. The *S*-operator encodes the action of the interaction given by the potential *V*. It is customary to give the relation $\psi^{\mathrm{out}}=S\psi^{\mathrm{in}}$ either on the momentum or in the energy representation. In the first case, *S* is a function of the momentum, S(k), in the second, of the energy, S(E).
    Under some causality conditions [1,2], the function S(k) can be analytically continued to a meromorphic function on the whole complex plane. Resonances appear as pairs of poles on the lower half of the complex plane, with the same imaginary part and the real parts equal in modulus, and with opposite signs.
    In the energy representation and under the same causality conditions, the *S*-operator is represented by a function S(E), meromorphic on a two-sheeted Riemann surface. Here, resonances are pairs of complex conjugate poles on the second sheet, located at the points $E_{R}\pm i\Gamma /2$, where $E_{R}>0$ and Γ have the same meaning as above, i.e., resonance energy and width.
    In principle, these resonance poles may have any multiplicity. However, although models with double pole resonances has been constructed [12], in most cases the multiplicity is equal to one.
    This definition usually matches with the physical definitions given above, although this is not always the case [13]. Models with resonance poles on the analytic continuation of the *S*-operator, not being escorted by a bump in the cross section with equal resonance energy and width have been constructed. Analogously, models having a bump in the cross section without the corresponding pole on the *S*-operator also exist (see [5]). Also, *S*-operators admitting an analytic continuation with weird properties have also been considered [14,15,16].
    Generally speaking, physicists prefer this definition to the next.
  - As we said before, in order to have resonances, we need a Hamiltonian pair $\left\{H_{0},H=H_{0}+V\right\}$, where the *V* is the potential responsible of the creation of a resonance. Since physical Hamiltonians are semibounded in non-relativistic quantum mechanics, we may assume that the continuous spectrum of both $H_{0}$ and *H* coincides with the positive semiaxis $R^{+}\equiv \left[0,\infty \right)$. For simplicity, we may assume that this spectrum is simple (This is a technical detail that we are not going to explain here. See for instance [17]). We may also assume an absence of point and continuous singular spectrum (If not, just take the absolutely continuous part of the spectrum [17]).
    Now, consider that in the Hilbert space H on which both Hamiltonian act there exists a dense subspace D⊂H, such that the following functions, defined for any ψ∈D,
    (2) $$R_{0\psi }\left(\lambda \right):=\langle \psi |{\left(H_{0}-\lambda I\right)}^{-1}\psi \rangle \;,\;R_{\psi }\left(\lambda \right);=\langle \psi |{\left(H-\lambda I\right)}^{-1}\psi \rangle \;,$$
    where *I* is the identity operator and λ a complex number, admit an analytic continuation across the positive semiaxis. The functions (2) are called the *resolvent functions*. Assume that $R_{0\psi }\left(\lambda \right)$ were analytic at $z_{R}=E_{R}-i\Gamma /2$ for any ψ∈D, even if there existed a ψ∈D such that $R_{\psi }\left(\lambda \right)$ shows a pole at $z_{R}=E_{R}-i\Gamma /2$. Then, we say that the Hamiltonian pair $\left\{H_{0},H\right\}$ exhibits a resonance with resonance energy $E_{R}$ and width Γ [18].
    We have to underline that both functions $R_{0\psi }\left(\lambda \right)$ and $R_{\psi }\left(\lambda \right)$ are complex analytic with a branch cut on the positive semiaxis $R^{+}$, since the inverses ${\left(H_{0}-\lambda I\right)}^{-1}$ and ${\left(H-\lambda I\right)}^{-1}$ are not defined for λ in the spectrum of $H_{0}$ and *H*. Nevertheless, this analytic continuation is possible from the first to the second Riemann sheet on where the function S(E) is defined [1,18]. Resonance poles appear as complex conjugate on the second sheet, exactly as happens with poles of S(E).
    Up to our knowledge, there is not a thoroughly study of models for which resonances as poles of S(E) and resonance poles given by the resolvent functions coincide. This is true for some studied cases. As proven in [19], this happens for the case of the Friedrichs model, which will be mentioned later as a solvable model showing resonances.

In the basic formalism of standard non-relativistic quantum mechanics, quantum states are represented by either a vector state of by a density operator, depending on whether the state is pure or mixed, respectively. The first attempt to describe an unstable quantum state used a vector state represented by a square integrable wave function. Then, it was necessary to characterize those wave functions that provide an unstable quantum state. Experiments have shown that most unstable quantum states decay approximately exponentially. Thus, a wave function ψ may represent such a decaying state if the probability amplitude

(3) $$\left|\langle \psi |e^{-itH}\psi \rangle \right|$$

decays exponentially with time, or in other words, it is of the form $A\;e^{-\alpha t}$, with A,α>0.

However, this is never the case. Let us represent (3) in the energy representation, so that the wave functions have the energy as the variable. Since the Hamiltonian *H* is semibounded, with the above simplifying hypothesis according to which it has $R^{+}\equiv \left[0,\infty \right)$ as a simple purely absolutely continuous spectrum, then (3) becomes

(4) $$|\langle \psi |e^{-itH}\psi \rangle |=\int_{0}^{\infty }{\left|\psi \left(E\right)\right|}^{2}e^{-iEt}\;dE.$$

According to the Riemann-Lebesgue lemma [20], (4) has a limit when t⟼∞ and this limit is zero. However, we cannot obtain from (4) an exponential decay, at least for all times. For small times, a simple operation [5] shows that

(5) $$\frac{d}{dt}\;{\left|\langle \psi |e^{-itH}\psi \rangle \right|}^{2}=0\;.$$

This contradicts the exponential decay hypothesis, since at t=0, we have

(6) $${\left.\frac{d}{dt}A^{2}\;e^{-2\alpha t}\right|}_{t=0}=-2\alpha A^{2}\neq 0\;,$$

which shows that the decay cannot be exponential at very short times.

For very long times, the decay cannot be exponential either. This was shown by L. Khalfin [21] using a theorem by Paley and Wiener [22]. In this case and for large times (t⟼∞) [5]

(7) $$|\langle \psi |e^{-itH}\psi \rangle |\approx e^{-ct^{q}}\;,\;q<1\;,\;\;c>0\;.$$

Then, it is admissible to identify a quantum decaying state with those ψ for which (4) is approximately a decaying exponential for a large interval of time. This interval should be sufficiently large so as to include most of standard observations, since these observations show an exponential decay [23,24].

Since exponential decay seems to be the characteristics of many unstable quantum systems, it seems reasonable to split the decaying state ψ into a sum of two contributions, such as $\psi =\psi_{G}+\psi_{B}$. Here, $\psi_{G}$ has a purely exponential decay (*G* comes for Gamow) and $\psi_{B}$ is the contribution responsible for all the deviations of the exponential decay law. Here *B* stands for *background*, using the terminology suggested in [5]. The wave function in the coordinate representation $\psi_{G}$ has been investigated for some models and it is shown that it should behave asymptotically as an exponential, no general or particular results are known for the explicit form of $\psi_{B}$. Nevertheless, we may say that it cannot be normalizable, otherwise $\psi_{G}$ would have been normalizable, since $\psi_{G}=\psi -\psi_{B}$.

As suggested by Gamow [25] and Nakanishi [26,27], pure exponentially decaying state vectors, called *Gamow vectors*, $\psi_{D}$, should be Eigenvalues of the total Hamiltonian with Eigenvalues equal to the resonance poles, so as to contain the relevant resonance information such as the resonant energy $E_{R}$ and the mean life as determined by the width Γ. Thus, for a resonance characterized by the pole $z_{R}=E_{R}-i\Gamma /2$, we define the Gamow vector as the solution of the following Eigenvalue equation:

(8) $$H\psi_{D}=\left(E_{R}-i\Gamma /2\right)\psi_{D},$$

so that

(9) $$e^{-itH}\psi_{D}=e^{-iE_{R}t}e^{-\Gamma t/2}\;\psi_{D}\;,$$

which implies that the time evolution to the future of the Gamow vector $\psi_{D}$ is purely exponential. The subindex *D* stands for *decaying*.

Then, a difficulty arises. The total Hamiltonian *H* is self adjoint and, hence, cannot have complex Eigenvalues on a Hilbert space. This difficulty is solved by extending the Hilbert space to a rigged Hilbert space, also called a Gelfand triplet. This will be explained in the next section.

The idea of the present review is to give a state of the art of some results on quantum unstable states, particularly after the work by the authors. Obviously, a more ambitious project would yield to the need of writing a whole book. Therefore, we are discussing here some particular topics such as the use of rigged Hilbert spaces to define rigorously the Gamow vectors and their properties in Section 2. Also in Section 2, we briefly describe the basis Friedrichs model as an exactly solvable model for resonances. In Section 3, we use a different mathematical model to show that Gamow states can be neither pure nor mixtures, but another different kind of object which was already used in quantum physics in a different context. We go to Section 4 to construct coherent Gamow states for the one dimensional hyperbolic Pöschl-Teller potential. Other considerations are exposed in Section 5. In Section 6, we propose a possible explanation of the Loschmidt echo based on a formulation using Gamow states. This paper closes with some concluding remarks and an Appendix A.

## 2. Rigged Hilbert Spaces and Gamow Vectors

In the previous section, we have defined the Gamow vectors as Eigenvectors of the total Hamiltonian with complex Eigenvalues, something incompatible with either the Hamiltonian being self adjoint or with the fact that the Eigenvectors belong to a Hilbert space. We can solve this dilemma by enlarging the Hilbert space, so that the Eigenvectors belong to a bigger space. This can be done with the use of rigged Hilbert spaces (RHS), also called Gelfand triplets.

Let H be an infinite dimensional separable Hilbert space. A RHS is a triplet of spaces,

(10) $$\Phi \subset H\subset \Phi^{\times }\;,$$

where, Φ is a dense subspace of H endowed with a locally convex topology finer than the topology inherited by the Hilbert space topology. In particular, this means that the canonical injection j:Φ⟼H, j(ψ)=ψ, ∀ψ∈Φ is continuous. The space $\Phi^{\times }$ is the *antidual* of Φ, which is the space of all continuous antilinear (Antilinearity means that if $F\in \Phi^{\times }$, ϕ,ψ∈Φ and α,β are complex numbers, one has that

$$F\left(\alpha \phi +\beta \psi \right)=\alpha^{*}\;F\left(\phi \right)+\beta^{*}\;F\left(\psi \right)\;,$$

where the star denotes complex conjugation. The antilinearity is used instead the linearity because it is covenient to match with the Dirac notation in quantum mechanics [28]. Antilinearity has essentially the same properties than linearity for practical purposes, such as the characterization of continuity for functionals.) functionals on Φ. Then, $\Phi^{\times }$ is endowed with a topology compatible (There are three of these topologies on $\Phi^{\times }$: strong, weak and McKey [29]. Nevertheless, this is a technical issue without real implications in our discussion.) with the dual pair $\left\{\Phi ,\Phi^{\times }\right\}$. This implies that any ψ∈H defines a unique continuous antilinear functional $F_{\psi }\in \Phi^{\times }$, given by $F_{\psi }\left(\phi \right):=\langle \phi |\psi \rangle$ [30], where 〈−|−〉 is the scalar product on H and the action of any $F\in \Phi^{\times }$ on ϕ∈Φ is denoted as F(ϕ), or equivalently 〈ϕ|F〉. We shall use the latter more often. The topology inherited by H from $\Phi^{\times }$ is coarser (has less open sets or zero neighbourhoods) than the Hilbert space topology, so that the canonical injection $j:H⟼\Phi^{\times }$ is continuous.

One of the main reasons for the interest in the RHS formalism is its usefulness for rigorously grounding the Dirac formalism. This construction is mainly based on the *Gelfand-Maurin theorem* [31,32]. Here, we express it in two separated parts, the first part states:

*Let H be a self adjoint operator on an infinite dimensional separable Hilbert space H, with domain D. Then, there exists a RHS of the form (10) with Φ⊂D, such that HD⊂D, i.e., Hψ∈D, for all ψ∈D and H is continuous with the topology on Φ.*

This is the crucial part for the purpose of defining the Gamow vectors for resonances. The point is that we may extend *H* to the antidual $\Phi^{\times }$ with continuity using the following *duality formula*:

(11) $$\langle H\psi |F\rangle =\langle \psi |HF\rangle \;,\;\forall \;\psi \in \Phi \;,\;\forall \;F\in \Phi^{\times }\;.$$

It results that $HF\in \Phi^{\times }$. In addition, *H* is linear and continuous on $\Phi^{\times }$ when endowed the latter with a topology compatible with the dual pair $\left\{\Phi ,\Phi^{\times }\right\}$. Note that we are using the same notation for the operator *H* and for the extension of *H* to $\Phi^{\times }$.

The second part of the Gelfand-Maurin Theorem may be stated as follows:

*Under some conditions for the topological structure of Φ, such as nuclearity (Another technical issue that we do not want to discuss here. For the details, see [33].), there exists a measure dμ on the spectrum of H, σ(H), such that:*

- For almost all (with respect to the measure dμ) λ∈σ(H), there exists a $F_{\lambda }\in \Phi^{\times }$ such that $HF_{\lambda }=\lambda F_{\lambda }$. It is customary the write $F_{\lambda }\equiv |\lambda \rangle$.
- The operator *H* admits a spectral decomposition of the form
  (12) $$\langle \phi |H\psi \rangle =\int_{\sigma (H)}\lambda \;\langle \phi |\lambda \rangle \langle \lambda |\psi \rangle \;d\mu \left(\lambda \right)\;,\;\forall \;\phi ,\psi \in \Phi \;,d\mu \left(\lambda \right)\;,$$
  where $\langle \lambda |\psi \rangle ={\langle \psi |\lambda \rangle }^{*}=:\psi \left(\lambda \right)$.

This second part of the Gelfand-Maurin theorem has the following implications:

1. Almost all λ∈σ(H) verify a Eigenvalue relation of the kind A|λ〉=λ|λ〉, which extends the Eigenvalue equation valid for the point spectrum to all spectrum. This is particularly important when *H* has an absolutely continuous spectrum [17]. The only difference is that, while the Eigenvectors with Eigenvalue in the continuous spectrum are vectors in H, the Eigenvectors $|\lambda \rangle \in \Phi^{\times }$ and |λ〉∉H.
2. If we omit the arbitrary ϕ,ψ∈Φ, we may write, for all $n\in N_{0}$,
  (13) $$H^{n}=\int_{\sigma (H)}\lambda^{n}\;|\lambda \rangle \langle \lambda |\;d\mu \left(\lambda \right)\;.$$
  For n=0, we obtain a spectral representation of the canonical injection $I:\Phi ⟼\Phi^{\times }$,
  (14) $$I=\int_{\sigma (H)}|\lambda \rangle \langle \lambda |\;d\mu \left(\lambda \right)\;.$$
  This canonical injection is continuous with respect to the topologies on Φ and $\Phi^{\times }$.
3. There exists a unitary mapping $U:H⟼L^{2}\left(\sigma \left(H\right),d\mu \right)$, such that $UHU^{-1}$ is the multiplication operator on $L^{2}\left(\sigma \left(H\right),d\mu \right)$. For each ψ∈Φ, Uψ=〈λ|ψ〉=ψ(λ). We have a new RHS of the form $U\Phi \subset L^{2}\left(\sigma \left(H\right),d\mu \right)\subset {\left(U\Phi \right)}^{\times }$. This new RHS is a concrete realization of (10), where the elements of the Hilbert space are functions and the elements of the antidual ${\left(U\Phi \right)}^{\times }$ are generalized functions in the sense of Gelfand [30].

Some additional interesting literature on RHS and their applications in quantum theory can be found in [34,35,36,37,38,39,40,41,42,43,44,45,46,47,48,49,50]. We do not pretend to be exhaustive.

### 2.1. Gamow Vectors

As we have previously commented, each resonance is characterized by a pair of poles of the analytic extension of the *S*-matrix in the energy representation S(E). These poles are located at the points $z_{R}=E_{R}-i\Gamma /2$ and its complex conjugate $z_{R}^{*}=E_{R}+i\Gamma /2$. These poles are usually simple, although poles of multiplicity bigger than one are also possible. A construction of an *S*-matrix containing double poles has been made in [12]. These poles have some particular implications in relation with the use of Gamow vectors, so that we are considering here resonances given by simple poles only, just for simplicity in our exposition.

Due to the fact that a resonance is given by a pair of poles and not a single one, along the Gamow vector defined in (8) and (9), we define the *growing* Gamow vector, $\psi_{G}$, as a vector having the following properties:

(15) $$H\psi_{G}=\left(E_{R}+i\Gamma /2\right)\;\psi_{G}\;,\;e^{-itH}\;\psi_{G}=e^{-iE_{R}t}\;e^{\Gamma /2}\;\psi_{G}\;,$$

so that $\psi_{G}$ grows exponentially with time. For this reason, we index this Gamow vector with letter *G*, meaning growing.

Now, let us make a list on the properties of the Gamow vector corresponding to a single resonance with resonance poles at $E_{R}\pm i\Gamma /2$ [51,52,53]:

- The Gamow vectors belong to the duals of respective RHS, $\Phi_{\pm }\subset H\subset \Phi_{\pm }^{\times }$, with $\psi_{D}\in \Phi_{+}^{\times }$ and $\psi_{G}\in \Phi_{-}^{\times }$ [53] (The convention of signs is the opposite in [52]. Nevertheless, we have considered convenient to use the present convention.). The total Hamiltonian *H* is extended by duality to the antiduals $\Phi_{\pm }^{\times }$ (11). Then, the compatibility between the self adjointness of *H* and the presence of Eigenvectors with complex Eigenvalues is explained by the fact that these Eigenvectors do not belong to the Hilbert space H.
- In the Introduction, we have mentioned the existence of a background, there represented by the vector state $\psi_{B}$, and responsible for the deviations of the exponential law. Recall that $\psi =\psi_{G}+\psi_{B}$, then, since $\psi \in H\subset \Phi_{+}$, we infer that $\psi_{B}\in \Phi_{+}$. It is rather obvious that another background vector must exist in $\Phi_{-}^{\times }$, due to the symmetry properties both RHS, $\Phi_{\pm }\subset H\subset \Phi_{\pm }^{\times }$.
- Using the simplification according to which *H* has a simple absolutely continuous spectrum $R^{+}\equiv \left[0,\infty \right)$, we may represent the RHS $\Phi_{\pm }\subset H\subset \Phi_{\pm }^{\times }$ using a triplet where H is represented by $L^{2}\left(R^{+}\right)$, and $\Phi_{\pm }$ are represented by respective spaces of analytic functions at least on a half plane. The most used representation for $\Phi_{\pm }$ is given by the following spaces
  (16) $$S\cap H_{\mp }^{2}{|}_{R^{+}}\;,$$
  where:
  (i) S is the Schwartz space of all indefinitely differentiable functions that converge to zero faster than the inverse if any polynomial.
  (ii) $H_{-}^{2}$ is the space of Hardy functions on the open lower half plane. These functions, $f_{-}\left(z\right)$, are analytic in the lower half plane with the property that
    (17) $$\underset{y>0}{\sup}\int_{-\infty }^{\infty }{\left|f_{-}\left(x-iy\right)\right|}^{2}\;dx<\infty \;.$$
    The functions of their boundary values on the whole real line R, $f_{-}\left(x\right)$, are square integrable and uniquely determine the function $f_{-}\left(z\right)$ as defined on the whole open half plane and vice-versa, $f_{-}\left(z\right)$ determines uniquely the boundary function $f_{-}\left(x\right)$ [54,55,56]. Also, f(z) can be determined by its boundary values on the positive semiaxis $R^{+}$ [57].
  (iii) In (16), the functions are restricted to the positive semiaxis [0,∞), so that the functions are considered as complex functions of positive real variable.
  (iv) The construction of the topology on (16) comes from the topology of the Schwartz space S through a procedure explained in [52,53].
  It is possible to construct a representation without this restriction to the positive semiaxis. In this representation, the Gamow vectors are normalizable (although outside the domain of *H*), so that they must be considered as members of the antidual spaces and have the Breit-Wigner energy distribution (1). However, this construction is not unitarily equivalent to $\Phi_{\pm }\subset H\subset \Phi_{\pm }^{\times }$ [52]. The Hardy functions on the upper half plane are defined analogously and have the same properties.
- The procedure for the construction of $\Phi_{\pm }\subset H\subset \Phi_{\pm }^{\times }$ goes as follows: The spectral theorem [17] gives a unitary operator $U:H⟼L^{2}\left(R^{+}\right)$, such that $UHU^{-1}$ is the multiplication operator on $L^{2}\left(R^{+}\right)$. Then, construct $\Phi_{\pm }$ as
  (18) $$\Phi_{\pm }:=U^{-1}\;\left[S\cap H_{\mp }^{2}{|}_{R^{+}}\right]\;.$$
  Once we have the topology on (16), we transport this topology to $\Phi_{\pm }$ by the unitary mapping $U^{-1}$.
  *H* is continuous on both $\Phi_{\pm }$, and $H\Phi_{\pm }\subset \Phi_{\pm }$. Then, we may extend *H* by continuity to $\Phi_{\pm }^{\times }$ by duality using (11). Gamow vectors are defined as taking full advantage of the analyticity properties of Hardy functions and we show Eigenvectors of this extension of *H* to the antiduals with the Eigenvalues given in (8) and (15).
- The interest of using spaces of Hardy functions in (16) is the following: Equation (9) is only valid for positive values of time, while the second relation in (15) is only valid for negative values of time. This result has several important consequences. The time evolution of the decaying Gamow vector, $\psi_{D}$, is defined for positive values of time, which is compatible with the idea that this vector represents the exact exponentially decaying part of a resonance. Otherwise, the time evolution of the growing Gamow vector, $\psi_{G}$, increases from −∞<t<0, which means that it *decays to the past*. This growing Gamow vector represents the same resonance as the decaying Gamow vector and it represent the same phenomenon. One is the time reversal of the other [58]. This construction is just the point of departure of another interesting formalism, the *time asymmetric quantum mechanics* that we do not intend to explain here. For details, see [59,60,61,62,63].

### 2.2. The Friedrichs Model

The original Friedrichs model is an exactly solvable toy model with one resonance [64,65]. It is noteworthy that this simple model shows all features of resonance theory, including the existence of scattering Møller operators, *S*-matrix, etc. Resonance poles defined with the resolvent and resonance poles as singularities of the function S(E) coincide [19]. The Friedrichs model has been generalized so as to describe more complex models showing resonances, although not all of the new approaches are exactly solvable due to their increasingly complexity [12,66,67,68,69,70,71,72,73]. Even for a construction of resonances in relativistic quantum field theory [74,75], the literature on the subject is far from being complete. In any case, they give a comprehensible idea on how resonance phenomena behaves on the quantum world [76].

The construction of the most basic Friedrichs model is the following. As any quantum system showing resonances, we have a Hamiltonian pair, $\left\{H_{0},H\right\}$. We shall assume that the free Hamiltonian $H_{0}$ possesses a simple (non-degenerate) absolutely continuous spectrum coinciding with the positive semiaxis, $R^{+}\equiv \left[0,\infty \right)$, plus a non-degenerate bound state immersed in the continuous. In the energy representation, we have the following spectral decomposition of $H_{0}$,

(19) $$H_{0}=\omega_{0}\;|1\rangle \langle 1|+\int_{0}^{\infty }\omega \;|\omega \rangle \langle \omega |\;d\omega \;,$$

where:

(i) |1〉 is a Eigenvector of $H_{0}$ with Eigenvalue $\omega_{0}>0$. This Eigenvector belongs to the Hilbert space domain of $H_{0}$ and, therefore, it represents a bound state, $H_{0}\;|1\rangle =\omega_{0}\;|1\rangle$.
(ii) Each of the |ω〉 is an Eigenvector of $H_{0}$ with Eigenvalue $\omega \in R^{+}$, $H_{0}\;|\omega \rangle =\omega \;|\omega \rangle$, the absolutely continuous spectrum of $H_{0}$. These Eigenvectors do not belong to the Hilbert space, but to the antidual of a RHS $\Phi \subset H\subset \Phi^{\times }$, with H the Hilbert space where $H_{0}$ and *H* act. The details of the construction of this RHS may be seen in [76].

The total Hamiltonian is a sum of two terms, $H=H_{0}+\lambda V$, where

(20) $$V=\int_{0}^{\infty }f\left(\omega \right)\;[|1\rangle \langle \omega |+|\omega \rangle \langle 1|]\;d\omega \;.$$

Here, f(ω) is a real (It may also be complex, although this would imply the need of make slight changes on the expression of the potential *V* in (20) [76].) square integrable function on the spectrum of $H_{0}$, called the *form factor* (It should have some additional properties as discussed in [19]. In particular, ${\left|f\left(\omega \right)\right|}^{2}$ should admit an analytic continuation.), and λ a real parameter. Observe that *V* intertwines bound state and continuous spectrum of $H_{0}$. In the limit λ⟼0, *H* goes to $H_{0}$.

In order to search for resonance poles, it is customary [19] to apply the definition of resonance poles given above in terms of the resolvent operator. If we define the complex function η(z) as

(21) $$\frac{1}{\eta (z)}:={\langle 1|\left(H-zI\right)}^{-1}|1\rangle \;,$$

we obtain

(22) $$\eta \left(z\right)=z-\omega_{0}-\lambda^{2}\int_{0}^{\infty }\frac{{\left|f\left(\omega \right)\right|}^{2}}{z-\omega }\;d\omega \;.$$

Under some mild conditions on the form factor [19], the function η(z) is a complex analytic function with two important features: (i) It has the positive semiaxis $R^{+}\equiv \left[0,\infty \right)$ as branch cut, and (ii) it has two complex conjugate zeros, one located at

(23) $$z_{R}=\omega_{0}+\lambda^{2}\int_{0}^{\infty }\frac{{\left|f\left(\omega \right)\right|}^{2}}{z_{R}-\omega }\;d\omega -2\pi i\lambda^{2}{\left|f\left(z_{R}\right)\right|}^{2},$$

and its complex conjugate, $z_{R}^{*}$. The same result is obtained if we use the formalism of the *S*-matrix [19]. There are no other resonance poles.

The decaying $\psi_{D}$ Gamow vector has the following representation:

(24) $$\psi_{D}=|1\rangle +\int_{0}^{\infty }\frac{\lambda \;f(\omega )}{z_{R}-\omega +i0}\;|\omega \rangle \;d\omega \;.$$

We should remember that $\psi_{D}$ is a functional over a linear space of vectors Φ. Assume that ϕ∈Φ. Then, 〈ϕ|1〉 is well defined as scalar product of two vectors in the Hilbert space. Also, for each $|\omega \rangle \in \Phi^{\times }$, the expression 〈ϕ|ω〉 is well defined as a complex number, so that for $\omega \in R^{+}$, the function ϕ(ω):=〈ϕ|ω〉 is well defined (and it is square integrable on $R^{+}$). Then, the action of the functional $\psi_{D}$ on the arbitrary ϕ∈Φ is given by

(25) $$\langle \phi |\psi_{D}\rangle =\langle \phi |1\rangle +\lambda \int_{0}^{\infty }\frac{f(\omega )}{z_{R}-\omega +i0}\;\langle \phi |\omega \rangle \;d\omega \;.$$

The integral in (25) should be understood as the action of the distribution ${\left(z_{R}-\omega +i0\right)}^{-1}$ to the function f(ω)ϕ(ω) (This is $\lim_{\alpha \to 0}\int_{0}^{\infty }\frac{f(\omega )}{z_{R}-\omega +i\alpha }\;\phi \left(\omega \right)\;d\omega \;.$) (see [30]).

For the growing Gamow vector, we have [76]

(26) $$\psi_{G}=|1\rangle +\lambda \int_{0}^{\infty }\frac{f(\omega )}{z_{R}^{*}-\omega -i0}\;|\omega \rangle \;d\omega \;,$$

where the meaning of (26) is similar to (24). These Gamow vectors satisfy (8), (9) and (15) [76].

Since in a Hilbert space the absolutely continuous subspace of a given self adjoint operator, say $H_{0}$, and the subspace generated by the bound states of $H_{0}$ are mutually orthogonal, one may conclude that the following relations should hold:

(27) $$\langle 1|1\rangle =1\;,\;\langle \omega |1\rangle =\langle 1|\omega \rangle =0\;,\;\langle \omega |\omega^{\prime }\rangle =\delta \left(\omega -\omega^{\prime }\right)\;.$$

Relations (27) show that

(28) $$\langle \psi_{G}|\psi_{D}\rangle =1+\lambda^{2}\int_{0}^{\infty }\frac{{\left|f\left(\omega \right)\right|}^{2}}{{\left(z_{R}-\omega +i0\right)}^{2}}\;d\omega ={\langle \psi_{D}|\psi_{G}\rangle }^{*}\;,$$

which, in principle, converges (The distribution ${\left(z_{R}-\omega +i0\right)}^{-2}$ may be considered as the derivative of ${\left(z_{R}-\omega +i0\right)}^{-1}$, in a distributional sense.) depending on the function f(ω). However, a similar operation shows that both $\langle \psi_{D}|\psi_{D}\rangle$ and $\langle \psi_{G}|\psi_{G}\rangle$ cannot be defined in a same manner, since a product of distributions of the kind ${\left(z_{R}-\omega -i0\right)}^{-1}{\left(z_{R}-\omega +i0\right)}^{-1}$ is not defined as a tempered distribution.

## 3. Gamow States as Functionals on an Algebra of Operators

Since the purely exponential part of a resonance can be described by a pair of Gamow vectors, or just one since the other one is just the time reversal of the other, one may assume that a Gamow state should be a pure state, as happens in ordinary quantum mechanics, with states described by normalizable vectors or wave functions. However, this point of view has some inconveniences. First of all, Gamow states are not normalizable, so that they do not fit exactly in the ordinary concept of quantum state. In addition, Gamow states describe unstable quantum states. Therefore, we cannot expect them to have zero entropy (For a study of the entropy associated with Gamow states see [77,78]) as happens with normalizable states.

To thoroughly investigate the nature of Gamow states, the RHS formulation is not sufficient. We propose another formulation based in the concept of quantum states as functionals over an algebra of observables. The origin of this formulation was the need to classify highly unstable quantum states within the context of statistical mechanics, now known as van Hove states or states with singular diagonal [79,80]. The original mathematical model for van Hove states was designed by Antoniou et al. [81] and was later used for various purposes [82,83,84,85,86].

It is interesting how this formalism may be applied to Gamow states. This discussion has been already given fundamentally in [87], and here we want to discuss its main points. In order to make the arguments simpler, we shall use a two Hamiltonian model $\left\{H_{0},H\right\}$ with the properties given in the previous sections, that means, its absolutely continuous spectrum of $H_{0}$ is simple, so that the absolutely continuous part of $H_{0}$ admits the following spectral decomposition [17]:

(29) $$H_{0}=\int_{0}^{\infty }E\;|E\rangle \langle E|\;dE\;,$$

with $H_{0}|E\rangle =E\;|E\rangle$, $E\in R^{+}$. Contrary to the situation for the Friedrichs model, we do not necessarily assume the existence of bound states for $H_{0}$. If the Møller operators exist and are asymptotically complete [88,89], we define $\Omega_{+}:=\Omega_{\mathrm{OUT}}$, $\Omega_{-}:=\Omega_{\mathrm{IN}}$, and

(30) $$|E^{+}\rangle :=\Omega_{+}\;|E\rangle \;|E^{-}\rangle =\Omega_{-}\;|E\rangle \;.$$

The kets $|E^{\pm }\rangle$ live in duals spaces of a RHS. The details can be found in previous publications [84,87]. Then, the perturbed Hamiltonian $H=H_{0}+V$ (or at least its absolutely continuous part) admits two spectral decompositions:

(31) $$H=\int_{0}^{\infty }E\;|E^{\pm }\rangle \langle E^{\pm }|\;dE\;,$$

with $H|E^{\pm }\rangle =E\;|E^{\pm }\rangle$, $E\in R^{+}$. Here, the sign plus stand for *outgoing* and the sign minus for *incoming*. Note that $H=\Omega_{\pm }H_{0}\Omega_{\pm }^{†}$, where we just consider the absolutely continuous part of both $H_{0}$ and *H*.

All the above kets satisfy the following “product” relations for $E,E^{\prime }\in R^{+}$ [84]

(32) $$\langle E|E^{\prime }\rangle =\delta \left(E-E^{\prime }\right)\;,\;\langle E^{\pm }|{E^{\prime }}^{\pm }\rangle =\delta \left(E-E^{\prime }\right)\;.$$

We say that the operators $O^{\pm }$ are *compatible* with *H* if they admit the following respective spectral decompositions [84]:

(33) $$O^{\pm }=\int_{0}^{\infty }O\left(E\right)\;|E^{\pm }\rangle \langle E^{\pm }|\;dE+\int_{0}^{\infty }dE\int_{0}^{\infty }dE^{\prime }\;O\left(E,E^{\prime }\right)\;|E^{\pm }\rangle \langle {E^{\prime }}^{\pm }|\;,$$

where O(E) and $O(E,E^{\prime })$ belong to spaces of test functions, so they admit analytic continuations on both variables independently.

The operators satisfying (33) form linear spaces, respectively. Under a proper choice of the functions under the integral signs in (33), the linear spaces are also algebras, here denoted as $A^{\pm }$. Taking into account relations (32), the product of two operators $O_{i}^{\pm }\in A^{\pm }$, with i=1,2, is given by:

(34) $$O_{1}^{\pm }\;O_{2}^{\pm }=\int_{0}^{\infty }O_{1}\left(E\right)\;O_{2}\left(E\right)\;|E^{\pm }\rangle \langle E^{\pm }|\;dE+\int_{0}^{\infty }dE\int_{0}^{\infty }dE^{\prime }\;O_{1}\left(E,E^{\prime }\right)\;O_{2}\left(E,E^{\prime }\right)\;|E^{\pm }\rangle \langle {E^{\prime }}^{\pm }|\;.$$

Note that the algebras $A^{\pm }$ are isomorphic.

In order to simplify the notation, and for later purposes, we define

(35) $$|E^{\pm }):=|E^{\pm }\rangle \langle E^{\pm }\left|\;,\;\right|{EE^{\prime }}^{\pm }):=|E^{\pm }\rangle \langle {E^{\prime }}^{\pm }|\;,$$

so that

(36) $$O^{\pm }=\int_{0}^{\infty }O\left(E\right)\;|E^{\pm })\;dE+\int_{0}^{\infty }dE\int_{0}^{\infty }dE^{\prime }\;O\left(E,E^{\prime }\right)\;\left|{EE^{\prime }}^{\pm }\right)\;.$$

Depending on the choice of the space of functions O(E), the algebras $A^{\pm }$ can have an identity. If we allow the function O(E)=1 for all values of *E*, then, we may define respective identities of $A^{\pm }$ as

(37) $$I^{\pm }:=\int_{0}^{\infty }dE\;\left|E^{\pm }\right)\;.$$

It is also convenient to define an involution on the algebras $A^{\pm }$. We define the *adjoint* of $O^{\pm }$ as

(38) $${\left(O^{\pm }\right)}^{†}=\int_{0}^{\infty }dE\;O^{*}\left(E\right)\;(E^{\pm }|+\int_{0}^{\infty }dE\int_{0}^{\infty }dE^{\prime }\;O^{*}\left(E^{\prime },E\right)\;\left({EE^{\prime }}^{\pm }\right|\;,$$

where the star means complex conjugation. The transposition of variables on the function under the double integral in (38) should be highlighted.

With the purpose of defining functionals on the algebras $A^{\pm }$, it is convenient to use the following conventions for $E,E^{\prime },w,w^{\prime }\in R^{+}$:

(39) $$\left(E^{\pm }|w^{\pm }\right)=\delta \left(E-w\right)\;,\;\left({EE^{\prime }}^{\pm }|{ww^{\prime }}^{\pm }\right)=\delta \left(E-w\right)\delta \left(E^{\prime }-w^{\prime }\right)\;,\;\left({EE^{\prime }}^{\pm }|w\right)=\left(w|{EE^{\prime }}^{\pm }\right)=0\;.$$

In particular, for any $O^{\pm }\in A^{\pm }$, we have

(40) $$\left(E^{\pm }|O^{\pm }\right)=O\left(E\right)\;,\;\left({EE^{\prime }}^{\pm }|O^{\pm }\right)=O\left(E,E^{\prime }\right)\;.$$

Relation (40) give a first example of functionals over the algebras $A^{\pm }$ as we see next.

### 3.1. Functionals over the Algebras

Relations (40) provide a tool for the construction of functionals on a topological algebra $A^{\pm }$ (an algebra endowed with a topology compatible with the algebraic structure). We recall that a functional *f* over an algebra A is a linear mapping f:A⟼C, continuous with respect to the topology on A.

Functionals over $A^{\pm }$ may be written in the following form, respectively:

(41) $$\rho^{\pm }:=\int_{0}^{\infty }dE\;\rho \left(E\right)\;(E^{\pm }|+\int_{0}^{\infty }dE\int_{0}^{\infty }dE^{\prime }\rho \left(E,E^{\prime }\right)\;\left({EE^{\prime }}^{\pm }\right|\;,$$

where ρ(E) is a functional over the space of functions O(E) and $\rho (E,E^{\prime })$ is a functional over the space of functions $O(E,E^{\prime })$. Taking into account the expression (38), the action of $\rho^{\pm }$ on $O^{\pm }$ is given by

(42) $$\left(\rho^{\pm }|O^{\pm }\right)=\int_{0}^{\infty }dE\;\rho \left(E\right)\;O\left(E\right)+\int_{0}^{\infty }dE\int_{0}^{\infty }dE^{\prime }\;\rho \left(E,E^{\prime }\right)\;O\left(E,E^{\prime }\right)\;.$$

The choice of the generalized functions (This term has been borowed from Gelfand, see [30,31]). ρ(E) and $\rho (E,E^{\prime })$ depends on the spaces of functions O(E) and $O(E,E^{\prime })$, respectively, and on the chosen topologies for these spaces of functions. After (40), it should be clear that $\left(E^{\pm }\right|$ is a functional with

(43) $$\rho \left(E\right)=\delta \left(E-w\right)\;,\;\mathrm{and}\;\rho \left(E,E^{\prime }\right)\equiv 0\;,$$

and $\left({EE^{\prime }}^{\pm }\right|$ is a functional with

(44) $$\rho \left(E\right)\equiv 0\;,\;\mathrm{and}\;\rho \left(E,E^{\prime }\right)=\delta \left(E-w\right)\delta \left(E^{\prime }-w^{\prime }\right)\;.$$

In what follows, we make some comments about the construction of the spaces of functions for O(E) and $O(E,E^{\prime })$ and their respective topologies. The idea of this construction has been suggested in [84] and a more thorough implementation will appear in a future publication. Here, we give the main ideas about the structure of these algebras of functions.

We choose as the space of functions O(E), $C^{\infty }\left(R^{+}\right)$, the space of continuous complex functions on the interval E∈[0,∞). These functions are bounded on all compact subsets of $R^{+}$. Let us define the topology on $C^{\infty }\left(R^{+}\right)$ using the following set of seminorms (A seminorm, p(−), on a vector space *X* is a mapping x⟼p(x)∈C, for all x∈X, such that p(x)≤0, p(λx)=|λ|p(x), for all λ∈C and x∈X and finally p(x+y)≤p(x)+p(y) for x,y∈X. Families of seminorms determine locally convex topologies on vector spaces over the complex (or even real) field): For any compact set $K\subset R^{+}$, we have

(45) $$p_{K}\left(O\left(E\right)\right):=\underset{E\in K}{\sup}\left|O\left(E\right)\right|\;.$$

This topology induces on $C^{\infty }\left(R^{+}\right)$ a metrizable and complete topology [90]. A sequence of functions $O_{n}\left(E\right)\subset C^{\infty }\left(R^{+}\right)$ converges to a function $O\left(E\right)\in C^{\infty }\left(R^{+}\right)$ if and only if for any compact set $K\subset R^{+}$, we have that

(46) $$p_{K}\left(O_{n}\left(E\right)-O\left(E\right)\right)⟼0\;.$$

Completeness means that any Cauchy sequence of functions $O_{n}\left(E\right)\subset C^{\infty }\left(R^{+}\right)$ converges to some function $O\left(E\right)\in C^{\infty }\left(R^{+}\right)$. $O_{n}\left(E\right)$ is Cauchy if for any ε>0, there exists a natural *N* such that if p,q>N, one has for any compact set $K\subset R^{+}$:

(47) $$p_{K}\left(O_{p}\left(E\right)-O_{q}\left(E\right)\right)<\varepsilon \;.$$

The choice of the algebra of functions $O(E,E^{\prime })$ that we propose in here is more involved. First, let us define the Schwartz spaces $S(R^{\pm })$ as the spaces of functions $f^{\pm }:R^{\pm }⟼C$, where $R^{-}\equiv \left(-\infty ,0\right]$, such that:

(i) They are differentiable at all points and at all orders.
(ii) The value of these functions and all their derivatives at the origin is zero.
(iii) They and all their derivatives go to zero at the infinity faster than the inverse of any polynomial.

The topology on these spaces is given by the following family of seminorms. For $f^{\pm }\left(E\right)\in S\left(R^{\pm }\right)$, one defines [17]:

(48) $$p_{r,s}\left(f^{\pm }\left(E\right)\right):=\underset{E\in R^{\pm }}{\sup}\left|E^{r}\;D^{s}\;f^{\pm }\left(E\right)\right|\;,\;r,s=0,1,2,\ldots \;,\;D=\frac{d}{d}E\;.$$

Since all functions in the Schwartz space are square integrable, we may define the alternative set of seminorms on the spaces $S(R^{\pm })$:

(49) $$q_{r,s}\left(f^{\pm }\left(E\right)\right):=\left|\right|E^{r}\;d^{s}\;f^{\pm }\left|\right|\;,\;r,s=0,1,\ldots \;,$$

where the norm in (49) is the standard norm on $L^{2}\left(R\right)$. Both family of seminorms on the spaces $S(R^{\pm })$ are equivalent in the sense that provide these spaces with the same topology.

In order to construct the algebra of functions of the kind $O(E,E^{\prime })$, we need to obtain the spaces of the Fourier transforms of functions in $S(R^{\pm })$. We denote these spaces as $\Xi^{\pm }:=F\left[S\left(R^{\pm }\right)\right]$. These spaces have the following properties:

(i) The functions in both $\Xi^{\pm }$ are Schwartz functions, due to the properties of the Fourier transform [20].
(ii) Due to the Paley-Wiener Theorems [54,55,56,91], the functions on $\Xi^{\pm }$ are Hardy functions in $H^{\mp }$, respectively. This analyticity property is important in order to define the Gamow functionals.

The space of functions is given by the tensor product,

(50) $$\Xi^{-}⊗\Xi^{+}\;.$$

This space is endowed with a tensor product locally convex topology that we do not describe here due to technical complexities, which are unnecessary in this presentation and will be published in a more technical article.

A second option is the use of the spaces $D(R^{\pm })$ instead of the spaces $S(R^{\pm })$. These are spaces of Schwartz functions which vanish outside a compact set contained in $R^{\pm }$, respectively. These spaces have a non-metrizable strict inductive limit topology [90]. The interest of these spaces is that their respective Fourier transforms, $Z^{\pm }:=F\left[S\left(R^{\pm }\right)\right]$ are analytically continued to entire functions and still Hardy on a half plane, following the Paley-Wiener theorem. Thus, another option for the space of functions $O(E,E^{\prime })$ is

(51) $$Z^{-}⊗Z^{+}\;.$$

The spaces of functions given by (50) and (51) are suitable for the construction of $A^{-}$. However, for $A^{+}$, we use instead,

(52) $$\Xi^{+}⊗\Xi^{-}\;,\;Z^{+}⊗Z^{-}\;.$$

### 3.2. The Gamow Functionals

First of all, let us recall the notion of a state on any algebraic formulation of quantum mechanics. Let us consider an algebra A with involution, $O⟼O^{†}$, and identity, *I*. By definition [92,93], a state over an algebra A of this kind is a linear functional f:A⟼C, such that:

(i) *f* is positive, i.e., for any O∈A, one has that $f(O^{†}\;O)\geq 0$.
(ii) *f* is normalized, i.e., f(I)=1, where *I* is the identity in A.
(iii) If A were endowed with a topology compatible with the algebraic structure, *f* should be continuous with respect to this topology and the usual topology on the complex plane C.

Assume that we have a resonance with resonance pole in the energy representation at $z_{R}=E_{R}-i\Gamma /2$ and its complex conjugate. Let us consider the generalized function (distribution) $\delta_{z_{R}}⊗\delta_{z_{R}^{*}}$ that act on $O(E,E^{\prime })$ in either the space (50) or (51) as

(53) $$\left(\delta_{z_{R}}⊗\delta_{z_{R}^{*}}|O\left(E,E^{\prime }\right)\right):=O\left(z_{R},z_{R}^{*}\right)\;,$$

and its extension by linearity and completeness with respect to the topology on either (50) or (51). Analogously, the generalized function $\delta_{z_{R}^{*}}⊗\delta_{z_{R}}$ acts on $O(E,E^{\prime })$ in either of the spaces (52) as

(54) $$\left(\delta_{z_{R}^{*}}⊗\delta_{z_{R}}|O\left(E,E^{\prime }\right)\right):=O\left(z_{R}^{*},z_{R}\right)\;.$$

Then, let us define the following functional on $A^{-}$:

(55) $$\rho_{G}:=\int_{0}^{\infty }dE\;\delta \left(E-E_{R}\right)\;(E^{-}|+\int_{0}^{\infty }dE\int_{0}^{\infty }dE^{\prime }\;\delta_{z_{R}}⊗\delta_{z_{R}^{*}}\;\left({EE^{\prime }}^{-}\right|\;,$$

and the equivalent functional on $A^{+}$:

(56) $$\rho_{D}:=\int_{0}^{\infty }dE\;\delta \left(E-E_{R}\right)\;(E^{-}|+\int_{0}^{\infty }dE\int_{0}^{\infty }dE^{\prime }\;\delta_{z_{R}^{*}}⊗\delta_{z_{R}}\;\left({EE^{\prime }}^{-}\right|\;.$$

Here the subindices *G* and *D* stand for *growing* and *decaying*, as we shall justify soon. Thus, $\rho_{G}$ and $\rho_{D}$ as in (55) and (56) receive the respective names of *growing Gamow functional (or state)* and *decaying Gamow functional (or state)*.

We need to show that $\rho_{D}$ is a state on $A^{-}$ and $\rho_{G}$ is a state on $A^{+}$. Linearity is obvious, so that both are linear functionals or simply functionals over their respective algebras. Since we have not specify the topology, we will not prove continuity due to the need of some technicalities. Also, we need some mathematical subtleties concerning Hardy functions to show that [84,87]

(57) $$\left(\rho_{G}|{\left(O^{+}\right)}^{†}\;O^{+}\right)=|O\left(E_{R}\right){|}^{2}+{\left|O\left(z_{R},z_{R}^{*}\right)\right|}^{2}\geq 0\;,$$

which proves the positivity of $\rho_{D}$. In addition, $\rho_{D}$ is normalized:

(58) $$(\rho_{G}|I^{-})=1\;,$$

where $I^{-}$ is the identity on $A^{-}$. This comes easily from (37) and (55). Therefore, $\rho_{D}$ is a state on $A^{-}$. Similar arguments show that $\rho_{D}$ is a functional on $A^{+}$.

Let us study some of the properties of these states. Let $\rho^{\pm }$ be states over the algebras $A^{\pm }$. An $O^{\pm }\in A^{\pm }$ is an observable if and only if ${\left(O^{\pm }\right)}^{†}=O^{\pm }$.

When one defines Gamow states using Gamow vectors, one finds a serious difficulty in defining the mean value of an observable *O* on the Gamow state. The reason is that in general $\langle \psi_{D}|O|\psi_{D}\rangle$ or $\langle \psi_{G}|O|\psi_{G}\rangle$ are not defined, even if the observable *O* is the Hamiltonian *H* [94,95,96,97]. The average of an observable $O^{\pm }\in A^{\pm }$ on an arbitrary state $\rho^{\pm }$, and on the Gamow states can always be defined as

(59) $$\left(\rho^{\pm }|O^{\pm }\right)\;,\;\left(\rho_{G}|O^{-}\right)\;,\;\left(\rho_{D}|O^{+}\right)\;,$$

where $\left(\rho^{\pm }|O^{\pm }\right)$ was given in (42).

A typical observable is the Hamiltonian *H*, which in the present formulation has the form (31). Note that after (32), we may have the natural powers of *H* in the following form:

(60) $$H^{n}=\int_{0}^{\infty }dE\;E^{n}\;\;|E^{\pm }\rangle \langle E^{\pm }|=\int_{0}^{\infty }dE\;E^{n}\;\left|E^{\pm }\right)\;.$$

Therefore,

(61) $$\left(\rho_{G}|H^{n}\right)=\left(\rho_{D}|H^{n}\right)=E_{R}^{n}\;,$$

where $E_{R}>0$ is the real part of a resonance pole. The same result has been obtained using different notions for n=1 in [94].

Go back to (55) and (56), which are the sum of two contributions. By convention, we call regular term the summand with one integral and singular term to that with two integrals. Thus, let us write (55) and (56), respectively, as

(62) $$\rho_{D}=\rho_{DR}+\rho_{DS}\;,\;\rho_{G}=\rho_{GR}+\rho_{GS}\;,$$

where the subindices *R* and *S* stand for *regular* and *singular*, respectively. Conventionally, the expressions of the Gamow functionals and their splits hold for an origin of times, t=0, or the time at which the constitution of the decaying state has been completed and the decay starts [1,98,99].

Let us give the path to find the time evolution for the Gamow functionals, where we omit some technical details that will blur the general idea. First of all, since the time evolution is given by the total Hamiltonian *H*, if $O^{\pm }$ is the observable at time t=0, the observable at time *t* should be

(63) $$\begin{array}{c} e^{itH}\;O^{\pm }\;e^{-itH}=\int_{0}^{\infty }dE\;O\left(E\right)\;e^{itH}\;\;|E^{\pm }\rangle \langle E^{\pm }|\;e^{-itH}\\ +\int_{0}^{\infty }dE\int_{0}^{\infty }dE^{\prime }\;O\left(E,E^{\prime }\right)\;e^{itH}\;|E^{\pm }\rangle \langle {E^{\prime }}^{\pm }|\;e^{-itH}\;. \end{array}$$

We need to obtain $e^{itH}\;|E^{\pm }\rangle$. In general, the duality formula (11) may be extended to any operator *A* under the condition that AΦ⊂Φ. If ψ∈Φ and $e^{-itH}\psi \in \Phi$ for some value of *t*, then, the duality formula

(64) $$\langle e^{-itH}\psi |F\rangle =\langle \psi |e^{itH}\;F\rangle$$

gives the action of $e^{itH}$ on an arbitrary functional $F\in \Phi^{\times }$ for this value of *t*.

The functionals $|E^{\pm }\rangle$ act on spaces of test vectors. We may always assume that these are spaces of functions depending on the energy (This is another technicality that we do not want to explain here. See for instance [53] or [50], where this issue is treated with more generality.) *E*. If ψ(E) is a wave function depending on the energy, we have $e^{itH}\psi \left(E\right)=e^{itE}\;\psi \left(E\right)$, so that (64) reads in this case

(65) $$\langle \psi^{\pm }\left(E\right)|e^{-itH}|E^{\pm }\rangle =\langle e^{itH}\;\psi^{\pm }\left(E\right)|E^{\pm }\rangle =e^{-itE}\;\langle \psi^{\pm }\left(E\right)|E^{\pm }\rangle \;,$$

where the minus sign right after the second equal sign comes from the antilinear character of the functionals $|E^{\pm }\rangle$. If we omit the arbitrary test functions $\psi^{\pm }\left(E\right)$ in (65), we finally have

(66) $$e^{-itH}\;|E^{\pm }\rangle =e^{-itE}\;|E^{\pm }\rangle \;.$$

It is necessary to remark that the space of test functions must be invariable under the action of $e^{itH}$ for convenient values of *t*, in order to (66) be well defined. We may construct these spaces so that (62) be valid for all values of *t* or for *t* either (Typically when the space of test functions is a Hardy space on a half plane [52,53].) on $R^{+}$ or in $R^{-}$.

Then, (62) becomes

(67) $$e^{itH}\;O^{\pm }\;e^{-itH}=\int_{0}^{\infty }dE\;O\left(E\right)\;|E^{\pm }\rangle \langle E^{\pm }|+\int_{0}^{\infty }dE\int_{0}^{\infty }dE^{\prime }\;O\left(E,E^{\prime }\right)\;e^{it(E-E^{\prime })}\;|E^{\pm }\rangle \langle {E^{\prime }}^{\pm }|\;.$$

Due to the construction of the functions $O(E,E^{\prime })$ using Hardy spaces on a half plane, we have that if t<0,

(68) $$\begin{array}{c} O\left(E,E^{\prime }\right)\in \Xi^{-}⊗\Xi^{+}⟹O\left(E,E^{\prime }\right)\;e^{it(E-E^{\prime })}\in \Xi^{-}⊗\Xi^{+}\;, \end{array}$$

(69) $$\begin{array}{c} O\left(E,E^{\prime }\right)\in Z^{-}⊗Z^{+}⟹O\left(E,E^{\prime }\right)\;e^{it(E-E^{\prime })}\in Z^{-}⊗Z^{+}\;. \end{array}$$

However, if t>0, we have

(70) $$\begin{array}{c} O\left(E,E^{\prime }\right)\in \Xi^{+}⊗\Xi^{-}⟹O\left(E,E^{\prime }\right)\;e^{it(E-E^{\prime })}\in \Xi^{+}⊗\Xi^{-}\;, \end{array}$$

(71) $$\begin{array}{c} O\left(E,E^{\prime }\right)\in Z^{+}⊗Z^{-}⟹O\left(E,E^{\prime }\right)\;e^{it(E-E^{\prime })}\in Z^{+}⊗Z^{-}\;. \end{array}$$

Relations (68) and () are not correct for any t>0 and (70) and () are not correct for any t<0 due to an argument concerning the properties of Hardy functions on a half plane [52].

The time evolution for the Gamow functionals comes after the following duality formula: If t<0,

(72) $$\begin{array}{cc} \left(e^{-itH}\;\rho_{G}\;e^{itH}|O^{-}\right) & =(\rho_{G}|e^{itH}\;O^{-}\;e^{-itH})\\ & =\int_{0}^{\infty }dE\;\delta \left(E-E_{R}\right)\;O\left(E\right)+\int_{0}^{\infty }dE\int_{0}^{\infty }dE^{\prime }\;\delta_{z_{R}}⊗\delta_{z_{R}^{*}}\;O\left(E,E^{\prime }\right)\;e^{it(E-E^{\prime })}\\ & =O\left(E_{R}\right)+O\left(z_{R},z_{R}^{*}\right)\;e^{it(z_{R}-z_{R}^{*})}\\ & =\left(\rho_{GR}|O^{-}\right)+e^{t\Gamma }\;\left(\rho_{GS}|O^{-}\right)\;. \end{array}$$

If we omit the arbitrary $O^{-}\in A^{-}$, we conclude that if t<0

(73) $$\rho_{G}\left(t\right)=e^{-itH}\;\rho_{G}\;e^{itH}=\rho_{GR}+e^{t\Gamma }\;\rho_{GS}\;.$$

Thus $\rho_{G}\left(t\right)$ has two parts, the regular part that do not evolve and the singular part that *grows* exponentially from t=−∞ to t=0. The time evolution of $\rho_{G}\left(t\right)$ for positive values of *t* is not defined if we choose the functions $O(E,E^{\prime })$ as defined before. This is why we call the Gamow state $\rho_{G}$ the *growing Gamow state*.

Analogously, for positive values t>0, we have that

(74) $$\rho_{D}\left(t\right)=\rho_{DR}+e^{-t\Gamma }\;\rho_{DS}\;.$$

As for the growing Gamow state, the time evolution (74) is not defined for negative values of time. After (74), we understand why $\rho_{D}$ is called the *decaying Gamow state*.

Once, we have shown the functional character of $\rho_{G}$ and $\rho_{D}$, let us classify the states as is usually done in quantum physics. There are three types of states [81,84]. This classification needs the general form of state in the present context given by (41).

(i) *Pure states:*
  If there exists a square integrable function $\psi \left(E\right)\in L^{2}\left(R^{+}\right)$ such that
  (75) $$\rho \left(E\right)={\left|\psi \left(E\right)\right|}^{2}\;,\;\rho \left(E,E^{\prime }\right)=\psi^{*}\left(E\right)\;\psi \left(E^{\prime }\right)\;.$$
  Note that ρ(E,E)=ρ(E).
(ii) *Mixtures:*
  Just defined by the relation ρ(E)=ρ(E,E). Note that pure states are a particular case of mixtures. For mixtures we do not need the existence of a square integrable function satisfying (75).
(iii) *Generalized states:*
  All the others. This include the van Hove states with diagonal singular [79,80,81], characterized by ρ(E)≠ρ(E,E), where they are still regular functions. Here, we may include states where one or both of the functions ρ(E) or $\rho (E,E^{\prime })$ are generalized functions (distributions).

The conclusion of the present subsection is clear: Gamow states are not pure states, a wrong perception that may be captured by the representation of these states as vectors. In addition, this algebraic representation of Gamow states allows the definition of mean values of observables on Gamow states, a definition invalid with the use of Gamow vectors.

## 4. Coherent Gamow States

Coherent states were originally defined as minimal dispersion states. For the one dimensional quantum harmonic oscillator, the wave functions for these states are quite easily constructed making the orthonormal basis of Hermite functions [100]. In addition, the harmonic oscillator coherent states are eigenfunctions of the annihilation operator *a*, for all complex numbers. If $\psi_{n}\left(x\right)$, with $n\in N_{0}$, are the normalized Hermite functions, the coherent state for the annihilation operator with Eigenvalue z∈C is

(76) $$\psi \left(z,x\right)=e^{-{\left|z\right|}^{2}/2}\;\sum_{n=0}^{\infty }\frac{z^{n}}{\sqrt{n!}}\;\psi_{n}\left(x\right)\;,\;a\;\psi \left(z,x\right)=z\;\psi \left(z,x\right),$$

where the series converge in the sense of the norm on $L^{2}\left(R\right)$. This notion has been generalized to other systems, as Eigenvectors of an annihilation operator for all complex Eigenvalues, as may be seen in [101]. The number of publications on coherent states is enormous, just let us quote a few [101,102,103,104,105,106]. An important property satisfied by coherent states is a sort of resolution of the identity. If we represent by |z〉 the coherent state with a|z〉=z|z〉 and z∈C, then,

(77) $$I=\frac{1}{\pi }\int_{C}|z\rangle \langle z|\;dz\;,\;dz=dx\;dy\;,\;z=x+iy\;.$$

Is it possible to construct coherent states using Gamow states? This depends on what we understand by coherent Gamow states and may depend on specific models having resonances. Since Gamow states are states for resonances, we have to add one restriction: coherent Gamow states cannot be an overcomplete system for the whole space, but only for that sector containing resonance states. In addition, it is desirable that the model showing resonances be exactly solvable in order to perform formal manipulations with the due rigor.

In a recent article [107], coherent Gamow states have been introduced for the one dimensional hyperbolic Pöschl-Teller potential [108,109]. Both trigonometric and hyperbolic Pöschl-Teller potentials are part of a list of one dimensional exactly solvable potentials [110], which are used as a manageable approximation to other potentials. The one dimensional hyperbolic Pöschl-Teller Hamiltonian is

(78) $$H=-\frac{\hbar^{2}}{2m}\;\frac{d^{2}}{dx^{2}}-\frac{\hbar^{2}}{2m}\;\frac{\lambda (\lambda -1)}{\cosh^{2}x}\;,$$

where λ is a parameter. This parameter may be complex. With the choice λ=1/2+iℓ, with ℓ>0, the potential is a repulsive barrier and the Hamiltonian (78) is self-adjoint. This is our choice. This Hamiltonian is exactly solvable, has an infinite number of resonances and these resonances can be exactly determined [111]. The resonance poles are located at

(79) $$z_{R}\left(n\right)=E_{R}-i\;\frac{\Gamma }{2}=\frac{\hbar^{2}}{2m}{\left[k_{1}\left(n\right)\right]}^{2}\;,\;z_{R}^{*}\left(n\right)=E_{R}+i\;\frac{\Gamma }{2}=\frac{\hbar^{2}}{2m}{\left[k_{2}\left(n\right)\right]}^{2}\;,$$

with

(80) $$k_{1}\left(n\right)=\ell -i\left(n+\frac{1}{2}\right)\;,\;k_{1}\left(n\right)=-\ell -i\left(n+\frac{1}{2}\right)\;,\;n=0,1,\ldots \;.$$

Relations (79) and (80) give

(81) $$E_{R}=\frac{\hbar^{2}}{2m}\;\left(\ell^{2}-\gamma_{n}^{2}\right)\;,\;\Gamma =\frac{\hbar^{2}}{2m}\;4\ell \gamma_{n}\;,\;\gamma_{n}=n+\frac{1}{2}\;.$$

Let us assume that $\varphi_{n}^{D}$ and $\varphi_{n}^{G}$ are the deacying and growing Gamow vector, respectively, for the (n+1)-th resonance, i.e., $H\varphi_{n}^{D}=z_{R}\left(n\right)\varphi_{n}^{D}$ and $H\varphi_{n}^{G}=z_{R}^{*}\left(n\right)\varphi_{n}^{G}$. Ladder operators have been found for these Gamow vectors [111]. For the decaying Gamow vectors, $\varphi_{n}^{D}$, we have

(82) $$\begin{array}{c} B_{n}^{-}:=\cosh x\;\partial_{x}+\left(i\ell +n+\frac{1}{2}\right)\;\sinh x\;, \end{array}$$

(83) $$\begin{array}{c} B_{n}^{+}:=-\cosh x\;\partial_{x}+\left(i\ell +n+\frac{1}{2}\right)\;\sinh x\;, \end{array}$$

where $\partial_{x}$ denotes derivation with respect to *x*. Then, the operators $B_{n}^{-}$ and $B_{n}^{+}$ are annihilation and creation operators, respectively, for the sequence of decaying Gamow vectors in the following sense:

(84) $$B_{n}^{-}\;\varphi_{n}^{D}=\varphi_{n-1}^{D}\;,\;B_{n}^{+}\;\varphi_{n-1}^{D}=\varphi_{n}^{D}\;,\;B_{0}^{-}\;\varphi_{0}^{D}=0\;,\;n=1,2,\ldots \;.$$

For the growing Gamow vectors, we have

(85) $$\begin{array}{c} C_{n}^{-}:=-\cosh x\;\partial_{x}+\left(-i\ell +n+\frac{1}{2}\right)\;\sinh x\;, \end{array}$$

(86) $$\begin{array}{c} C_{n}^{+}:=\cosh x\;\partial_{x}+\left(-i\ell +n+\frac{1}{2}\right)\;\sinh x\;, \end{array}$$

so that

(87) $$C_{n}^{-}\;\varphi_{n}^{G}=\varphi_{n-1}^{G}\;,\;C_{n}^{+}\;\varphi_{n-1}^{G}=\varphi_{n}^{G}\;,\;C_{0}^{-}\;\varphi_{0}^{G}=0\;,\;n=1,2,\ldots \;.$$

In order to obtain the explicit form of the wave functions for the Gamow vectors, we use the last equations in (84) and (87), which are simple first order ordinary differential equations, for which the respective solutions are, save for a multiplicative constant, given by:

(88) $$\varphi_{0}^{D}\left(x\right)={\left(\cosh x\right)}^{i\ell +1/2}\;,\;\varphi_{0}^{G}\left(x\right)={\left(\cosh x\right)}^{-i\ell +1/2}\;.$$

The remaining wave functions are obtained by successive use of the operators $B_{n}^{+}$ and $C_{n}^{+}$ and have the form:

(89) $$\varphi_{n}^{D}\left(x\right)=P_{n}\left(\sinh x\right)\;\varphi_{0}^{D}\left(x\right)\;,\;\varphi_{n}^{G}\left(x\right)=Q_{n}\left(\sinh x\right)\;\varphi_{0}^{G}\left(x\right)\;,$$

where $P_{n}\left(\sinh x\right)$ and $Q_{n}\left(\sinh x\right)$ are polynomials of order *n* on sinhx.

We may consider the two linear spaces spanned by $\left\{\varphi_{n}^{D}\right\}$ and $\left\{\varphi_{n}^{G}\right\}$. On these spaces, we define the index-free operators, $B^{\pm }$ for the former and $C^{\pm }$ for the second one by

(90) $$B^{-}\;\varphi_{n}^{D}:=\sqrt{n}\;B_{n}^{-}\;\varphi_{n}^{D}\;,\;B^{+}\;\varphi_{n}^{D}:=\sqrt{n+1}\;B_{n}^{+}\;\varphi_{n}^{D}\;,$$

and

(91) $$C^{-}\;\varphi_{n}^{G}:=\sqrt{n}\;C_{n}^{-}\;\varphi_{n}^{G}\;,\;C^{+}\;\varphi_{n}^{G}:=\sqrt{n+1}\;C_{n}^{+}\;\varphi_{n}^{G}\;,$$

and extended by linearity to the spaces spanned by $\left\{\varphi_{n}^{D}\right\}$ and $\left\{\varphi_{n}^{G}\right\}$, respectively.

For any complex number z∈C, we want to find two collection of coherent states, $|z^{D}\rangle$ and $|z^{G}\rangle$ such that

(92) $$B^{-}\;|z^{D}\rangle =z\;|z^{D}\rangle \;,\;C^{-}\;|z^{G}\rangle =z\;|z^{G}\rangle \;.$$

Let us proceed with the construction of $|z^{D}\rangle$ [107]. It should have the form:

(93) $$|z^{D}\rangle =\sum_{n=0}^{\infty }c_{n}\;\varphi_{n}^{D}⟹B^{-}\;|z^{D}\rangle =\sum_{n=0}^{\infty }c_{n}\;\sqrt{n}\;\varphi_{n-1}^{D}=z\;\sum_{n=0}^{\infty }c_{n}\;\varphi_{n}^{D}\;,$$

which shows that

(94) $$c_{n}=\frac{c_{0}}{\sqrt{n!}}\;z^{n}\;.$$

The constant $c_{0}$ is arbitrary, so that we choose $c_{0}=1$. Same procedure gives $|z^{G}\rangle$. It remains to add that the series in (93) converge in the weak topology (This is a technicality, which we do not want to discuss in here.), a fact that has been proven in [107]. The same for the series that define $|z^{G}\rangle$.

### Resolutions of the Identity

In Ref. [87], we have shown that, in presence of resonances, the total Hamiltonian admits the following spectral decomposition:

(95) $$H=\sum_{n}z_{R}\left(n\right)\;|\varphi_{n}^{D}\rangle \langle \varphi_{n}^{G}|+\mathrm{background}\;,$$

where the *background* accounts for those effects that avoid a pure exponential decay, such as Zeno [5,10,112] or Khalfin [5,11,113] effects for very short and very large values of time, as well as the pollution by all kind of noise. On the spaces of resonances, we may consider just the first part, which in the case of the model under our consideration, we may write as:

(96) $$H=\sum_{n=0}^{\infty }z_{R}\left(n\right)\;|\varphi_{n}^{D}\rangle \langle \varphi_{n}^{G}|\;.$$

Recalling the triplets $\Phi_{\pm }\subset H\subset \Phi_{\pm }^{\times }$ defined in Section 2.1 and that $\varphi_{n}^{G}\in \Phi_{-}^{\times }$ and $\varphi_{n}^{D}\in \Phi_{+}^{\times }$, the expansion (95) is a continuous operator $H:\Phi_{-}⟼\Phi_{+}^{\times }$. Since $\Phi_{-}\subset \Phi_{+}^{\times }$ (Recall that $\Phi_{-}\subset H$ and $H\subset \Phi_{+}^{\times }$.), we have a (continuous) canonical injection $I_{-}:=\Phi_{-}⟼\Phi_{+}^{\times }$, defined as

(97) $$I_{-}=\sum_{n=0}^{\infty }|\varphi_{n}^{D}\rangle \langle \varphi_{n}^{G}|\;.$$

Next, let us consider the following expression:

(98) $$\frac{1}{\pi }\int_{C}|z^{D}\rangle \langle z^{G}|\;e^{-{\left|z\right|}^{2}}\;dz\;,$$

with dz=dxdy and z=x+iy. Take arbitrary $\varphi^{\pm }\in \Phi_{\pm }$ and write

(99) $$\frac{1}{\pi }\int_{C}\langle \varphi^{+}|z^{D}\rangle \langle z^{G}|\varphi^{-}\rangle \;e^{-{\left|z\right|}^{2}}\;dz\;.$$

Then, after (93) and (94) we have

(100) $$\langle \varphi^{+}|z^{D}\rangle =\sum_{n=0}^{\infty }\frac{z^{n}}{\sqrt{n!}}\;\langle \varphi^{+}|\varphi_{n}^{G}\rangle =\sum_{n=0}^{\infty }\frac{z^{n}}{\sqrt{n!}}\;\varphi^{+}\left(z_{R}\left(n\right)\right)\;.$$

The second identity in (100) is due to the representation of the space $\Phi_{+}$ as a space of analytic functions. Thus, $\langle \varphi^{+}|\varphi_{n}^{G}\rangle$ is equal to the value at $z_{R}\left(n\right)$ of the analytic function $\varphi^{+}\left(z\right)$, that represents the vector $\varphi^{+}$ in the space of analytic functions.

Analogously,

(101) $$\langle z^{G}|\varphi^{-}\rangle =\sum_{m=0}^{\infty }\frac{{\left(z^{*}\right)}^{m}}{\sqrt{m!}}\;\langle \varphi_{n}^{G}|\varphi^{-}\rangle =\sum_{m=0}^{\infty }\frac{{\left(z^{*}\right)}^{m}}{\sqrt{m!}}\;{\left[\varphi^{-}\left(z_{m}^{*}\right)\right]}^{*}\;.$$

Due to the choice of the analytic functions being Hardy on a half plane [52,53], integral (99) converges. In addition, a simple manipulation [107] gives

(102) $$\frac{1}{\pi }\int_{C}\langle \varphi^{+}|z^{D}\rangle \langle z^{G}|\varphi^{-}\rangle \;e^{-{\left|z\right|}^{2}}\;dz=\sum_{n=0}^{\infty }\langle \varphi^{+}|\varphi_{n}^{D}\rangle \langle \phi_{n}^{G}|\varphi^{-}\rangle \;,$$

so that, if we omit the arbitrary $\varphi^{\pm }\in \Phi_{\pm }$, we conclude that

(103) $$\frac{1}{\pi }\int_{C}|z^{D}\rangle \langle z^{G}|\;e^{-{\left|z\right|}^{2}}\;dz=\sum_{n=0}^{\infty }|\varphi_{n}^{D}\rangle \langle \varphi_{n}^{G}|=I_{-}\;,$$

so that (98) is the identity (97). Analogously,

(104) $$\frac{1}{\pi }\int_{C}|z^{G}\rangle \langle z^{D}|\;e^{-{\left|z\right|}^{2}}\;dz=\sum_{n=0}^{\infty }|\varphi_{n}^{G}\rangle \langle \varphi_{n}^{D}|=I_{+}\;,$$

which is a sort of formal adjoint of (103). Clearly, the left hand sides of (103) and (104) are sorts of resolutions of the identity, similar to (98). The exponential term in the measure is essential, so that integrals like (99) make sense [107]. This measure has been introduced by Bargmann [114].

In addition, Gamow states may be considered as a particular example of pseudo-bosons, in some sense [107,115].

## 5. From Non-Commutativity to Commutativity

As is well known, there exists several approaches to the study of the classical limit of a quantum system, including decoherence and others [116]. In previous papers, we have discussed an alternative approach based on the evolution of the algebra of observables. From this point of view, a non-commutative algebra of observables evolves to a commutative algebra when time goes to infinite. However, this is not possible if the time evolution is unitary; therefore, we need to consider more general time evolutions. Approaches based in non-standard time evolution has previously been considered in several publications mostly by our group [53,86,116,117,118,119]. Here, we review a further generalization of non-unitary time evolution valid for quantum decaying systems, that we have introduced in a couple of previous publications [97,120].

We consider systems with *N* distinct resonances, where we drop any external interaction and limit ourselves to states of these resonances as combinations of Gamow states. From a strictly mathematical point of view, we may construct a scalar product on the space spanned by the Gamow states, although this product has no physical meaning. Some studies concerning physical properties of resonances [26,27,84,121,122,123] suggest that it is more convenient to introduce a pseudo-scalar metrics on this space, so that it has the structure of a Krein space. We may define observables as operators on this Krein space. It is the objective to study time evolution for these observables and show that the non-commutativity of these observables becomes commutativity (or approximate commutativity) for sufficiently large times.

To begin with our presentation, let us assume a quantum decaying system with *N* resonances. It is, however, true that many quantum models with resonances exhibit an infinite number of them, but all except for an infinite number of resonance poles have an imaginary part so large that make them unobservable. Recall that the imaginary part of a resonance pole is related with the inverse of the surviving time. Also, it may eventually happen that all except a finite number of resonance poles have very high energies, so that they fall into the relativistic frame and, therefore, out a context of non-relativistic quantum mechanics.

If the *N* resonance poles in the energy representation are located at the points $z_{1},\ldots ,z_{N}$ and their corresponding complex conjugates, the decaying Gamow vectors are labelled by

(105) $$|\psi_{1}^{D}\rangle \;,\ldots \;,|\psi_{N}^{D}\rangle \;,\;H|\psi_{i}^{D}\rangle =z_{i}\;|\psi_{i}^{D}\rangle \;,\;i=1,\ldots ,N\;.$$

The growing Gamow vectors are labelled by

(106) $$|\psi_{1}^{G}\rangle \;,\ldots \;,|\psi_{N}^{G}\rangle \;,\;H|\psi_{i}^{G}\rangle =z_{i}^{*}\;|\psi_{i}^{G}\rangle \;,\;i=1,\ldots ,N\;.$$

These Gamow vectors provide of corresponding spectral decompositions of the Hamiltonian in terms of non-hermitian components [97,124]:

(107) $$H=\sum_{i=1}^{N}z_{i}\;|\psi_{i}^{D}\rangle \langle \psi_{i}^{G}|+\mathrm{BGR}\;,$$

and

(108) $$H^{†}=\sum_{i=1}^{N}z_{i}^{*}\;|\psi_{i}^{G}\rangle \langle \psi_{i}^{D}|+{\mathrm{BGR}}^{*}\;.$$

The term BGR (background) includes all effects that do not affect Gamow states, such as Zeno and Khalfin effects and any kind of noise. Since we are only interested in Gamow states, we henceforth drop this term in both spectral decompositions (107) and (108). Note that both representations are non-hermitian and one is the formal adjoint of the other. Mathematically, these operators are continuous linear mappings: $H:\Phi_{-}⟼\Phi_{+}^{\times }$ and $H^{†}:\Phi_{+}⟼\Phi_{-}^{\times }$.

In the sequel, we consider the 2N-dimensional space $H^{G}$, spanned by the Gamow vectors (105) and (106). Then, we define a pseudometric on $H^{G}$ as the bilinear form that in the basis given by the 2N Gamow vectors is defined by the following matrix:

(109) $$A:=\left(\begin{array}{ccccccc} 0 & 1 & \ldots & \ldots & \ldots & \ldots & \ldots \\ 1 & 0 & \ldots & \ldots & \ldots & \ldots & \ldots \\ \ldots & \ldots & 0 & 1 & \ldots & \ldots & \ldots \\ \ldots & \ldots & 1 & 0 & \ldots & \ldots & \ldots \\ \ldots & \ldots & \ldots & \ldots & \ldots & \ldots & \ldots \\ \ldots & \ldots & \ldots & \ldots & \ldots & 0 & 1\\ \ldots & \ldots & \ldots & \ldots & \ldots & 1 & 0 \end{array}\right)\;,$$

so that $H^{G}$ is a Krein space.

The matrix *A* is a block diagonal matrix and all blocks are equal to the Pauli matrix $\sigma_{x}$. The pseudo-scalar product, (−|−), of two arbitrary vectors $|\psi \rangle ,|\varphi \rangle \in H^{G}$ is given by

(110) (ψ|φ):=〈ψ|A|φ〉,

so that,

(111) $$\left(\psi_{i}^{D}|\psi_{j}^{D}\right)=\left(\psi_{i}^{G}|\psi_{j}^{G}\right)=0\;,\;\left(\psi_{i}^{D}|\psi_{j}^{G}\right)=\left(\psi_{i}^{G}|\psi_{j}^{D}\right)=\delta_{ij}\;,$$

where $\delta_{ij}$ is the Kronecker delta. From (110), we note that if there existed a matrix *B* such that $B^{2}=A$, then, one would have that

(112) $$B|\psi_{i}^{D}\rangle =|\psi_{i}^{D})\;,\;\langle \psi_{i}^{G}|B=(\psi_{i}^{G}|\;,\;i=1,\ldots ,N\;.$$

This matrix *B* does exists, although it is not uniquely defined. One choice for *B* is the replacement of all blocks $\sigma_{x}$ in (109) by

(113) $${\left(-i\right)}^{1/2}\left(\begin{array}{cc} i\sqrt{2}/2 & \sqrt{2}/2\\ \sqrt{2}/2 & i\sqrt{2}/2 \end{array}\right)\;.$$

Then, with some natural amendments in the formalism, $|\psi_{i}^{D})$ and $|\psi_{j}^{G})$ may also represent the Gamow vectors. These amendments are simple. Just redefine the Hamiltonian *H* in (107), without the BGR term as

(114) $$H:=\sum_{i=1}^{N}z_{i}\;B\;|\psi_{i}^{D}\rangle \langle \psi_{i}^{G}|\;B=\sum_{i=1}^{N}z_{i}\;|\psi_{i}^{D})(\psi_{i}^{G}|\;,$$

so that

(115) $$H|\psi_{j}^{D})=z_{j}\;|\psi_{j}^{D})\;,\;\mathrm{and}\;H^{n}=\sum_{i=1}^{N}z_{i}^{n}\;|\psi_{i}^{D})(\psi_{i}^{G}|\;,\;n=1,2,\ldots \;.$$

Therefore, the formal action of *H* on the decaying Gamow vectors makes sense with the the introduction of the pseudometric. In addition, we have the following formal expression:

(116) $$e^{-itH}=\sum_{j=1}^{N}e^{-itz_{j}}\;|\psi_{j}^{D})(\;\psi_{j}^{G}|\;.$$

Now, we replace *B* in (112) by $C:=B^{†}$, so that (112) becomes

(117) $$|\psi_{i}^{G})=C\;|\psi_{i}^{G}\rangle \;,\;(\psi_{i}^{D}|C=\langle \psi_{i}^{D}|\;,\;i=1,\ldots ,N\;.$$

Thus, $H^{†}$ is given by

(118) $$H^{†}=C\left[\sum_{i=1}^{N}z_{i}^{*}\;|\psi_{i}^{G}\rangle \langle \psi_{i}^{D}|\right]C\;,$$

which implies that

(119) $$H^{†}|\psi_{j}^{G})=z_{j}^{*}\;|\psi_{j}^{G})\;,\;e^{-itH^{†}}=\sum_{j=1}^{N}e^{-itz_{j}^{*}}\;|\psi_{j}^{G})(\psi_{j}^{D}|\;,\;j=1,\ldots ,N\;.$$

We have some additional interesting results, all valid for j=1,…,N:

(120) $$H|\psi_{j}^{G})=0\;,\;H^{†}\left|\psi_{j}^{D}\right)=0\;,$$

(121) $$e^{-itH}\;|\psi_{j}^{D})=e^{-itz_{j}}\;|\psi_{j}^{D})\;,\;e^{-itH^{†}}\;|\psi_{j}^{G})=e^{-itz_{j}^{*}}\;\left|\psi_{j}^{G}\right)\;,$$

and

(122) $$e^{-itH}\;|\psi_{j}^{G})=0\;,\;e^{-itH^{†}}\;\left|\psi_{j}^{D}\right)=0\;.$$

Consequently, the operators *H* and $H^{†}$ act on a subspace of $H^{G}$ with dimension *N* and the operators $e^{-itH}$ and $e^{-itH^{†}}$ on its complementary subspace. Therefore, we need an extension of these operators to the whole $H^{G}$. This extension should be

(123) $$H=\sum_{i=1}^{N}z_{i}\;|\psi_{I}^{D})(\psi_{i}^{G}|+z_{i}^{*}\;|\psi_{I}^{G})(\psi_{i}^{D}|\;.$$

With this definition *H* is formally Hermitian. Using our previous results, we obtain the following for i=1,…,N:

(124) $$H|\psi_{i}^{D})=z_{i}\;|\psi_{i}^{D})\;,\;H^{†}|\psi_{i}^{G})=z_{i}^{*}\;\left|\psi_{i}^{G}\right)\;,$$

and

(125) $$H^{n}=\sum_{i=1}^{N}\left\{z_{i}^{n}\;|\psi_{i}^{D}\right)(\psi_{i}^{G}|+{\left(z_{i}^{*}\right)}^{n}\;\left|\psi_{i}^{G}\right)\left(\psi_{i}^{D}|\right\}\;.$$

However, we cannot assume that $e^{-itH}$ is the sum of (116) and the second equation in (119), as would have been the obvious formal consequence of (125). The reason has been explained with detail in [97]. If *O* were an arbitrary observable on the space $H^{G}$, then its time evolution should be given by O(t)=U(−t)OU(t). This gives undesirable contributions to O(t) that grow exponentially [97]. In order to avoid this problem, we have defined the time evolution operator on $H^{G}$ as

(126) $$U\left(t\right)=\sum_{i=1}^{N}e^{-itz_{i}}\;|\psi_{i}^{D})(\psi_{i}^{G}|+e^{itz_{i}^{*}}\;|\psi_{i}^{G})(\psi_{i}^{D}|\;,$$

which is formally Hermitian and time asymmetric. In addition, we have [97]

(127) $$U\left(t\right)U^{†}\left(t\right)=U^{2}\left(t\right)\;,$$

instead of $U\left(t\right)U^{†}\left(t\right)=I$. Here, the identity is

(128) $$I:=\sum_{i=1}^{N}\left\{|\psi_{i}^{D}\right)(\psi_{i}^{G}\left|+\right|\psi_{i}^{G})\left(\psi_{i}^{D}|\right\}\;.$$

Then, the time evolution of the observable *O* is O(t):=U(t)OU(t), with U(t) as in (126). The time evolution of a system with *N* resonances can be very complex, due to the presence of several different decay times. In order to illustrate the transition from non-commutative to commutative observables, we consider a system of one resonance $z=E_{R}-i\Gamma /2$. Let $O_{1}$ and $O_{2}$ be two observables on $H^{G}$, and let them evolve with the time evolution law governed by (126). Their commutator at time *t* is given by

(129) $$\begin{array}{cc} \left[O_{1}\left(t\right),O_{2}\left(t\right)\right] & =U\left(t\right)O_{1}U\left(t\right)U\left(t\right)O_{2}U\left(t\right)-U\left(t\right)O_{2}U\left(t\right)U\left(t\right)O_{1}U\left(t\right). \end{array}$$

Replacing the time evolution U(t), given in (126) with N=1, in the Equation (129), we obtain a sum of 16 terms. Each term is multiplied by an exponential decay with the same characteristic time 2Γ. Therefore,

(130) $$\left[O_{1}\left(t\right),O_{2}\left(t\right)\right]\propto e^{-2\Gamma t}.$$

The conclusion is that, in the limit as t⟼∞, the commutator between two operators on the space of Gamow vectors becomes zero. This fact could be seen as a kind of quantum classical transition for quantum unstable states for large values of time.

## 6. Irreversible Phenomena and Loschmidt Echo

Much has been discussed about time reversal and the arrow of time in relation to the problem of irreversibility (see [125,126]). It has been argued that the tendency of isolated physical systems to reach equilibrium implies an arrow of time. However, this becomes problematic as soon as one leaves the macroscopic world: the microscopic systems that make up the macroscopic systems are blind to any temporal direction. Although the traditional place of discussion of this problem has been classical statistical mechanics, the same argument naturally applies to quantum mechanics. In this case, what is observed is that the fundamental equation of the theory, the Schrödinger equation, is invariant under time reversal. So, it does not distinguish past-future direction from future-past direction.

This problem is particularly difficult since we cannot do experiments in the future-past direction of time. Therefore, the discussion has been developed mainly at a conceptual and formal level. However, some physical models have properties that allow the generation of experimental conditions that simulate a time reversal. For example, a spin system in the presence of a magnetic field *B* in the axis $\hat{z}$ has a Hamiltonian $H=\alpha BS_{z}$, where α is a coupling constant and $S_{z}$ is the spin operator in the direction $\hat{z}$. This Hamiltonian changes its sign when the direction of the magnetic field is changed. That is, if we change *B* by −B, *H* changes to −H, and the time evolution operator changes as follows

(131) $$U\left(t\right)=e^{-iHt}⟶e^{iHt}=U\left(-t\right).$$

Therefore, the effect of changing the sign of the magnetic field is mathematically the same as changing the sign of time. Within this type of system, experiments of the Loschmidt echo type can be set up with a scheme like the following:

(i) A quantum system with an initial state $|\psi (t_{0})\rangle$ is prepared at time $t_{0}=0$.
(ii) During a interval of time τ a magnetic field *B* is applied in such a way that the system evolves according to the evolution operator $U\left(\tau \right)=e^{-iH\tau }$. During this step the system is said to move “forward in time.”
(iii) Then, during the same interval of time τ, the magnetic field is reversed in such a way that the system evolves according to the evolution operator $U\left(-\tau \right)=e^{iH\tau }$. During this step the system is said to move “backwards in time".
(iv) The magnetic field is turned off and the initial state $|\psi (t_{0})\rangle$ and the final state $|\psi (t_{f})\rangle$, with $t_{f}=2\tau$, are compared.

According to the formalism of quantum mechanics, the evolution of the state throughout this experiment should be as follows [127,128]:

(132) $$|\psi \left(t_{0}\right)\rangle \to e^{-iH\tau }|\psi \left(t_{0}\right)\rangle \to e^{iH\tau }e^{-iH\tau }|\psi \left(t_{0}\right)\rangle =|\psi \left(t_{0}\right)\rangle =|\psi \left(t_{f}\right)\rangle .$$

This shows that the initial and final states should be exactly the same, which is consistent with thinking that the system moved forwards and backwards in time. In this way, the discrepancy between the initial and final state should be zero. However, the experiments carried out on spin systems located in the atomic nuclei of a crystal show that there are discrepancies between both states [129]. It was also observed that if τ is larger, then the discrepancy grows.

To study this phenomenon, the following magnitude is defined

(133) $$M=|\langle \psi \left(t_{0}\right)|\psi \left(t_{f}\right)\rangle {|}^{2}.$$

This is a measure of the discrepancy between the initial and final state and is often called Loschmidt echo.

The traditional way of incorporating equilibrium arrival, decoherence and the classical limit into the quantum description is based on considering open systems [116]. That is, the system of interest is considered to be in interaction with other quantum systems (environment). This explains the phenomenon, because when considering an open system that interacts with its environment, the state of the open system does not evolve according to the Schrödinger equation and, therefore, the evolution is not necessarily unitary. In the described experiment, it is also assumed that when the magnetic field is reversed, the sign of the Hamiltonian that governs the temporal evolution of the system is changed. However, if the considered system is subjected to an environment through an interaction, then, the reversal of the magnetic field will not have the effect of changing the sign of the total Hamiltonian, but will only change the sign of a part. Therefore, one can speak of an imperfect time reversal.

Following this line of reasoning, it can be said that the system evolves with a Hamiltonian $H_{0}$ when it evolves forward in time and with a slightly different one when it evolves backwards, i.e., $H_{0}-\Sigma$, where Σ is a perturbation. And for this reason, at the end of the process the system does not recover the initial state.

However, there are reasons to think that the introduction of the environment cannot explain the observations in some experiments and the existence of an “essential” irreversibility has been proposed [86,129,130,131,132,133,134,135,136,137]. The discussion about whether irreversibility should be attributed to the presence of an environment or to a fundamental characteristic of nature is beyond the scope of this article. However, as we shall see, the formalism presented in this article can be used for describing the phenomenon in both schemes.

On one hand, it is possible to interpret the Hamiltonian of the Equation (114) as describing a fundamental or intrinsic irreversibility of the system. However, on the other hand, it is also possible to interpret it as an effective Hamiltonian, that is, a Hamiltonian that includes the effects of the environment on the system. Leaving aside this philosophical debate, the application of the former formalism is very simple.

In the formalism based on the intrinsic irreversibility, the Hamiltonian is allowed to have complex Eigenvalues $z=E_{R}-i\Gamma /2$. Thus, the time evolution operator can be represented as $U\left(t\right)=e^{-iE_{R}t}e^{-\frac{\Gamma }{2}t}$. So, in the evolution operator, the real part $E_{R}$ of the Eigenvalue appears in a complex exponential and it is interpreted as the traditional energy of the system. While the imaginary part Γ appears in a decreasing exponential with characteristic time $2\Gamma^{-1}$. The introduction of decreasing exponential functions in the time evolution allows to account for the arrival at equilibrium, the decoherence and the classical limit [138].

Below we summarize an example published in a previous paper [139] that illustrates how the Gamow vector formalism can be applied to this particular problem. Then, we propose a model for the Loschmidt echo based on the previous four steps.

Let us consider a model with resonances, with the Hamiltonian *H* given by (123). Firstly, (i) we define the initial state of the system. We suppose that the system is in a superposition state that can be represented as

(134) $$|\psi \left(t_{0}\right))=\sum_{k=0}^{N}\alpha_{k}\;\left|\psi_{k}^{D}\right)\;,$$

where $\alpha_{k}\in C$.

Secondly, (ii) during an interval of time τ the system evolves “forward in time”according to the Hamiltonian

(135) $$H=\sum_{i=1}^{N}z_{i}\;|\psi_{i}^{D})(\psi_{i}^{G}|+z_{i}^{*}\;|\psi_{i}^{G})(\psi_{i}^{D}|\;,$$

with $z_{i}=E_{i}-\frac{i}{2}\Gamma_{i}$. Then, using Equation (126), we can compute the time evolution of the state given in (134),

(136) $$U\left(\tau \right)|\psi \left(t_{0}\right))=\sum_{i,k=1}^{N}e^{-i\tau z_{i}}\;\alpha_{k}|\psi_{i}^{D})\left(\psi_{i}^{G}|\psi_{k}^{D}\right)+e^{i\tau z_{i}^{*}}\alpha_{k}\;\left|\psi_{i}^{G}\right)\left(\psi_{i}^{D}|\psi_{k}^{D}\right)\;.$$

Considering equations (111), we have

(137) $$U\left(\tau \right)|\psi \left(t_{0}\right))=\sum_{k=1}^{N}\alpha_{k}e^{-i\tau z_{k}}\;\left|\psi_{k}^{D}\right)\;.$$

Thirdly, (iii) the magnetic field is reversed in such a way that a new Hamiltonian $H^{\prime }$ is obtained. In the standard approach, the new Hamiltonian $H^{\prime }$ is represented as a perturbation of the original Hamiltonian *H*. In our alternative model, the irreversibility is due to the decaying exponential factor related to the time evolution of the Gamow vectors. The action of reversing the magnetic field has the consequence that the real part of the Hamiltonian changes its sign, but the resonances remain the same. This is because resonances are related to internal degrees of freedom, and have nothing to do with the orientation of the system respect to the laboratory. Thus, the Hamiltonian $H^{\prime }$ has the following complex Eigenvalues:

(138) $$z_{i}^{\prime }=-E_{i}-\frac{i}{2}\Gamma_{i}=-\left(E_{i}+\frac{i}{2}\Gamma_{i}\right)=-z_{i}^{*}\;.$$

From Equation (126) we can compute the new time evolution operator

(139) $$U^{\prime }\left(t\right)=\sum_{i=1}^{N}e^{itz_{i}^{*}}\;|\psi_{i}^{D})(\psi_{i}^{G}|+e^{-itz_{i}}\;|\psi_{i}^{G})(\psi_{i}^{D}|\;,$$

and we can compute the time evolution of the state given in (137) after an interval of time τ

$$U^{\prime }\left(\tau \right)U\left(\tau \right)|\psi \left(t_{0}\right))=\sum_{i,k=1}^{N}\alpha_{k}e^{-i\tau z_{k}}e^{i\tau z_{i}^{*}}\;|\psi_{i}^{D})\left(\psi_{i}^{G}|\psi_{k}^{D}\right)+\alpha_{k}e^{-i\tau z_{k}}e^{-i\tau z_{i}}\;\left|\psi_{i}^{G}\right)\left(\psi_{i}^{D}|\psi_{k}^{D}\right)\;.$$

Considering equations (111), we have

(140) $$U^{\prime }\left(\tau \right)U\left(\tau \right)|\psi \left(t_{0}\right))=\sum_{k=1}^{N}\alpha_{k}e^{-i\tau \omega_{k}}e^{i\tau \omega_{k}^{*}}\;\left|\psi_{k}^{D}\right)\;.$$

Taking into account that $z_{k}=E_{k}-\frac{i}{2}\Gamma_{i}$ and Equation (138), we obtain

(141) $$U^{\prime }\left(\tau \right)U\left(\tau \right)|\psi \left(t_{0}\right))=\sum_{k=1}^{N}\alpha_{k}e^{-\tau \Gamma_{k}}\;\left|\psi_{k}^{D}\right)\;.$$

Finally, (iv) we compare the initial and final states using the Loschmidt echo,

(142) $$M\left(\tau \right)=(\psi \left(t_{0}\right)|U^{\prime }\left(\tau \right)U\left(\tau \right)|\psi \left(t_{0}\right))=\sum_{j=0}^{N}\alpha_{j}^{*}(\psi_{j}^{G}|\sum_{k=1}^{N}\alpha_{k}e^{-\tau \Gamma_{k}}\;|\psi_{k}^{D})=\sum_{k=1}^{N}{\left|\alpha_{k}\right|}^{2}e^{-\tau \Gamma_{k}}\;.$$

For simplicity, we consider a system with a single resonance. It can be shown that the Loschmidt echo has an exponential decay given by (see [139])

(143) $$M\left(\tau \right)\propto e^{-2\Gamma t}.$$

In addition, in the Heisenberg representation, it is possible to calculate the time evolution of the commutator between two observables $O_{1}$ and $O_{2}$ (see [97]),

(144) $$\left[O_{1}\left(t\right),O_{2}\left(t\right)\right]\propto e^{-2\Gamma t}\;.$$

This means that the commutator between two observables, initially incompatible, decays exponentially, and therefore, the observables become compatible in long times. This is the distinguishing feature of the classical limit. From expressions (143) and (144), we can conclude that there is a deep link between the irreversibility associated with the Loschmidt echo and the classical limit phenomenon.

## 7. Discussion and Conclusions

In the present article, we have reviewed in a compact and self-contained manner several notions concerning non-relativistic quantum unstable systems and the mathematics supporting these notions. To begin with, we recalled the notion of rigged Hilbert space (RHS) and its main applications. We gave a rigorous form to the Dirac formulation of quantum mechanics which allowed us to construct Gamow vectors. These vectors are the state vectors for the purely exponential decaying part of an unstable quantum state, also called scattering resonance. As is well known, resonances decay following an approximate exponential law, except for small and very high ranges of time. These ranges of time are practically unobservable in most experiments, so that a pure exponential decay law for a resonance is commonly accepted for most situations. The need to fix a vector state to describe this situation has been claimed since the beginning of quantum mechanics. Gamow vectors are Eigenvectors of the Hamiltonian with complex Eigenvalues that produce the decay.

We have very briefly reviewed the well known Friedrichs model as an exactly solvable model with resonances. Already within the context of the Friedrichs model, we observe that a kind of generalized scalar product between Gamow vectors is not possible and, therefore, we cannot rigorously define average values of observables on Gamow states.

To correct this problem, we have proposed a model in which Gamow vectors are functionals over a given algebra of operators. This model permits a consistent definition of averages of certain observables, such as the Hamiltonian, on Gamow states. In addition, this model shows that Gamow states are not pure states, thus getting rid of a misconception according to which Gamow states should be pure states, since they are represented by vectors and not density matrices. Gamow states are neither pure nor mixtures, but another kind of states very similar to diagonal singular states introduced for systems far from equilibrium. After this consideration, it makes sense the search for an approach for the entropy for unstable quantum systems based on Gamow states.

Due to a large interest in different kinds of quantum coherent states, we have shown that we may also construct coherent Gamow states. To this end, we have used an exactly solvable model having an infinite number of resonances and some mathematical properties of Hardy functions.

Transitions between a non-commutative algebra of observables to a commutative one is a main feature of the classical-quantum transitions. This is not possible using unitary evolution laws. Using solely the space spanned by Gamow vectors, i.e., ignoring short and very long time effects, we have shown that with an adequate definition of time evolution, we may get such a kind of transition over large values of time.

Finally, quantum systems with resonances permit an intrinsic description of some phenomena showing microscopic irreversibility, such as the Loschmidt echo, using Gamow states and their properties.

## Data Availability

Not applicable.

## Appendix A. A Model of Krein Space for Gamow Vectors

In the construction of Gamow vectors using spaces of Hardy functions on a half plane, one uses an intermediate RHS [52,53]. The construction goes as follows: Let $H_{\pm }\cap S$ be the intersection of the Schwartz space S with the space of Hardy functions on the upper half plane of the complex plane, with plus sign, and on the lower half plane, with minus sign. Due to the properties of these three spaces, one concludes that [52,53]

(A1) $$H_{\pm }\cap S\subset L^{2}\left(R\right)\subset {\left(H_{\pm }\cap S\right)}^{\times }$$

are two well defined RHS, where the topology on both $H_{+}\cap S$ and $H_{-}\cap S$ is the topology inherited from that on the Schwartz space S and the topology on the anti-duals could be either the strong or the weak topologies [17]. Consider $f_{\pm }\left(E\right)\in H_{\pm }\cap S$, so that $f_{\pm }^{\#}\left(E\right)={\left[f_{\pm }\left(E\right)\right]}^{*}\in H_{\mp }\cap S$. If $z_{R}=E_{R}-i\Gamma /2$ is a resonance pole, we have after the Titchmarsh theorem [54,55,56] that

(A2) $$f_{+}^{\#}\left(z_{R}\right)=-\frac{1}{2\pi i}\int_{-\infty }^{\infty }\frac{{\left[f_{+}\left(E\right)\right]}^{*}\;dE}{E-z_{R}}=\langle f_{+}^{\#}|\psi_{z_{R}}\rangle \;.$$

Analogously,

(A3) $$f_{-}^{\#}\left(z_{R}^{*}\right)=\frac{1}{2\pi i}\int_{-\infty }^{\infty }\frac{{\left[f_{-}\left(E\right)\right]}^{*}\;dE}{E-z_{R}^{*}}=\langle f_{-}^{\#}|\psi_{z_{R}^{*}}\rangle \;.$$

The functions

(A4) $$|\psi_{z_{R}}\rangle :=-\frac{1}{2\pi i}\;\frac{1}{E-z_{R}}\;\mathrm{and}\;|\psi_{z_{R}^{*}}\rangle :=\frac{1}{2\pi i}\;\frac{1}{E-z_{R}^{*}}$$

belong to [52,53]

(A5) $$|\psi_{z_{R}}\rangle \in {\left(H_{-}\cap S\right)}^{\times }\;,\;|\psi_{z_{R}^{*}}\rangle \in {\left(H_{+}\cap S\right)}^{\times }\;.$$

Observe that

(i) The functions (A4) are square integrable and, therefore, belong to $L^{2}\left(R\right)$.
(ii) These functions do not belong to the domain of the multiplication operator as $\frac{E}{E-z_{R}}$ and $\frac{E}{E-z_{R}^{*}}$ are not square integrable on R. Note that the multiplication operator on the energy representation is the Hamiltonian, under certain conditions on it [52,53] that we may assume without loosing nothing essential. Thus, the functions (A4) are out of the domain of the Hamiltonian. In addition, as one may directly check from the definition of Hardy function on a half plane, we have that $\frac{1}{E-z_{R}}\in H_{+}^{2}$ and $\frac{1}{E-z_{R}^{*}}\in H_{-}^{2}$.

Then, let us considered the two dimensional space spanned by the linearly independent (This independence is a reasonable Ansatz.) Gamow vectors $\{|\psi_{z_{R}}\rangle ,|\psi_{z_{R}^{*}}\rangle \}$ and the matrix

(A6) $$A=\left(\begin{array}{cc} 0 & 1\\ 1 & 0 \end{array}\right)\;.$$

Then,

(A7) $$\left(\psi_{z_{R}}|\psi_{z_{R}}\right):=\langle \psi_{z_{R}}\left|A\right|\psi_{z_{R}}\rangle =\frac{1}{{\left(2\pi i\right)}^{2}}\int_{-\infty }^{\infty }\frac{dE}{{\left(E-z_{R}\right)}^{2}}\;.$$

Note that

(A8) $$\int_{-\infty }^{\infty }\frac{dE}{{\left(E-z_{R}\right)}^{2}}=\int_{-\infty }^{\infty }\frac{1}{{\left(E-z_{R}^{*}\right)}^{*}}\;\frac{1}{E-z_{R}}\;dE=0\;,$$

since it is the scalar product of a function in $H_{-}^{2}$ times another in $H_{+}^{2}$, spaces which are orthogonal in $L^{2}\left(R\right)$ [55,56]. Thus, $(\psi_{z_{R}}|\psi_{z_{R}})=0$. Same for $(\psi_{z_{R}^{*}}|\psi_{z_{R}^{*}})=0$. On the other hand,

(A9) $$\left(\psi_{z_{R}}|\psi_{z_{R}^{*}}\right)=\langle \psi_{z_{R}}\left|A\right|\psi_{z_{R}^{*}}\rangle =\frac{1}{4\pi^{2}}\int_{-\infty }^{\infty }\frac{1}{{\left(E-z_{R}\right)}^{*}}\;\frac{1}{E-z_{R}}\;dE=\frac{1}{4\pi^{2}}\int_{-\infty }^{\infty }\frac{dE}{|E-z_{R}{|}^{2}}>0\;.$$

Note that the vectors (A4) are not normalized in $L^{2}\left(R\right)$. In order for these vectors to be normalized, we have to redefine them as

(A10) $$|\psi_{z_{R}}\rangle :=\sqrt{\frac{\Gamma }{2\pi }}\;\frac{1}{E-z_{R}}\;\mathrm{and}\;|\psi_{z_{R}^{*}}\rangle :=\sqrt{\frac{\Gamma }{2\pi }}\;\frac{1}{E-z_{R}^{*}}\;,$$

so that $(\psi_{z_{R}}|\psi_{z_{R}^{*}})=1$. Analogously, $(\psi_{z_{R}^{*}}|\psi_{z_{R}})=1$. So far, the situation goes exactly as described in Section 5.

However, if we have *N* resonances and we want to reproduce equations (111), we have to make some changes. Let us call the resonance poles $z_{1},\ldots ,z_{N}$, with $z_{i}=E_{i}-\Gamma_{i}/2$, and let $z_{i}^{*}$, with i=1,…,N, be their corresponding complex conjugate. Clearly, $\left(\psi_{z_{i}}|\psi_{z_{j}}\right)=\left(\psi_{z_{i}^{*}}|\psi_{z_{j}^{*}}\right)=0$, for i,j=1,…,N, and $\left(\psi_{z_{i}}|\psi_{z_{i}^{*}}\right)=\left(\psi_{z_{i}^{*}}|\psi_{z_{i}}\right)=1$, for i=1,…,N. However, with *A* as in (109), $(\psi_{z_{j}}|\psi_{z_{i}^{*}})\neq 0$ and, similarly, $(\psi_{z_{j}^{*}}|\psi_{z_{i}})\neq 0$ if i≠j. Thus, in order to reproduce (111), we have to define for i≠j:

(A11) $$\left(\psi_{z_{i}}|\psi_{z_{j}^{*}}\right)=\langle \psi_{z_{i}}|\psi_{z_{j}^{*}}\rangle =\frac{\sqrt{\Gamma_{i}\;\Gamma_{j}}}{2\pi }\int_{-\infty }^{\infty }\frac{1}{E-z_{i}^{*}}\;\frac{1}{E-z_{j}^{*}}\;dE=0\;,$$

so that in this particular case, we should omit the matrix *A* in order to obtain relations (111).
